# Identification and validation of *Aeluropus littoralis* reference genes for Quantitative Real-Time PCR Normalization

**DOI:** 10.1186/s40709-016-0053-8

**Published:** 2016-07-19

**Authors:** Seyyed Hamidreza Hashemi, Ghorbanali Nematzadeh, Gholamreza Ahmadian, Ahad Yamchi, Markus Kuhlmann

**Affiliations:** Genetics and Agricultural Biotechnology Institute of Tabarestan (GABIT), Sari Agricultural Sciences and Natural Resources University, PO Box 578, Sari, Iran; National Institute of Genetics Engineering and Biotechnology (NIGEB), Tehran, Iran; Department of Plant Breeding and Biotechnology, Gorgan University of Agricultural Sciences and Natural Resources, Gorgan, Iran; Research Group Abiotic Stress Genomics, Leibniz Institute of Plant Genetics and Crop Plant Research (IPK Gatersleben), OT Gatersleben Corrensstraße 3, 06466 Stadt Seeland, Germany

**Keywords:** Reference genes, qPCR, *Aeluropus littoralis*, Salt stress, Recovery condition, Halophyte, DNA contamination, rDNA, rRNA

## Abstract

**Background:**

The use of stably expressed genes as normalizers has crucial role in accurate and reliable expression analysis estimated by quantitative real-time polymerase chain reaction (qPCR). Recent studies have shown that, the expression levels of common housekeeping genes are varying in different tissues and experimental conditions. The genomic DNA contamination in RNA samples is another important factor that also influence the interpretation of the data obtained from qPCR. It is estimated that the gDNA contamination in gene expression analysis lead to an overestimation of the RNA transcript level. The aim of this study was to validate the most stably expressed reference genes in two different tissues of *Aeluropus littoralis*—halophyte grass at salt stress and recovery condition. Also, a qPCR-based approach for monitoring contamination with gDNA was conducted.

**Results:**

Ten candidate reference genes participating in different biological processes were analyzed in four groups of samples including root and leaf tissues, salt stress and recovery condition. To determine the most stably expressed reference genes, three statistical methods (geNorm, NormFinder and BestKeeper) were applied. According to results obtained, ten candidate reference genes were ranked based on the stability of their expression. Here, our results show that a set of four housekeeping genes (HKGs) e.g. *RPS3*, *EF1A*, *GTF* and *RPS12* could be used as general reference genes for the all selected conditions and tissues. Also, four set of reference genes were proposed for each tissue and condition including: *RPS3*, *EF1A* and *UBQ* for salt stress and root samples; *RPS3*, *EF1A*, *UBQ* as well as *GAPDH* for recovery condition; *U2SURP* and *GTF* for leaf samples. Additionally, for assessing DNA contamination in RNA samples, a set of unique primers were designed based on the conserved region of ribosomal DNA (rDNA). The universality, specificity and sensitivity of these primer pairs were also evaluated in Poaceae.

**Conclusions:**

Overall, the sets of reference genes proposed in this study are ideal normalizers for qPCR analysis in *A.littoralis* transcriptome. The novel reference gene e.g. *RPS3* that applied this study had higher expression stability than commonly used housekeeping genes. The application of rDNA-based primers in qPCR analysis was addressed.

**Electronic supplementary material:**

The online version of this article (doi:10.1186/s40709-016-0053-8) contains supplementary material, which is available to authorized users.

## Background

The use of wild plant species or their halophytic relatives has been considered in plant breeding programs for developing salt and drought tolerant crops [1]. In this view, several researchers focused on the *Aeluropuslittoralis* as a halophyte model for identification and isolation of the novel adaptation genes. *Aeluropuslittoralis* is a perennial monocot grass with the small haploid genome of 349 Mb (2n = 2X = 14) using the C_4_ mechanism for carbon fixation [2] that grows in dry salty areas or marshes [3]. *Aeluropuslittoralis* can survive where the water salinity is periodically high [4] and tolerate up to 1100 mM sodium chloride [5]. Therefore, *A.littoralis* can serve as valuable genetic resource for understanding the molecular mechanisms of stress-responses in monocots, and can potentially be used for improving tolerance to abiotic stresses in economically important crops [6]. Several morphological, anatomical, ecological, and physiological traits of *A*. *littoralis* have been investigated so far [5, 7, 8]. Meanwhile, a number of ESTs (expressed sequence tag), genes and promoters induced by the salt and drought stresses were isolated, sequenced and annotated at a molecular level [1, 3].

With the availability of genome and transcriptome sequence information in most plant species, the identification and characterization of stress-induced genes is more feasible now. Network analysis of transcriptome data might lead to better understanding of complex traits such as survival, growth and differentiation [9, 10]. Quantitative real-time polymerase chain reaction (qPCR) analysis is one of the most currently used approaches for measuring gene expression level [11]. The sensitivity, specificity and simplicity of this technique is incomparable with other methods such as Northern and in situ hybridization, RNase protection assays and semi-quantitative reverse transcription-polymerase chain reaction (RT-PCR) [12]. For accurate and reliable quantification of the gene expression, some important issues need to be considered when a qPCR approach is used. This includes variations in the amount of starting material, RNA quality and quantity, efficiency of cDNA synthesis, and PCR efficiency among different cells and tissues [13, 14]. Among various procedures that have been applied to minimize variability and maximize reproducibility, the internal control genes, often referred to as reference genes or housekeeping genes (HKGs) are most frequently used for normalization [15]. The expression level of an ideal reference gene should be constant across all respected cells, tissues and experimental conditions beside of its least expression variance between the group of samples analyzed [13]. Recent findings show that there is no universal reference gene (with high expression stability) for all biological questions addressed [10]. Based on the above-mentioned facts, valid reference genes for every organism should be verified for each tissue, condition and developmental stage. Fortunately, in most studies, only a limited number of tissues and treatments are examined, so probably one or more genes are stably expressed under that condition [13].

Until recently, the HKGs *18S rRNA*, *UBIQUITIN* (*UBQ*), *ACTIN* (*ACT*), *B*-*TUBULIN* (*TUB*), and glyceraldehyde-3-phosphate dehydrogenase (*GAPDH*), all involved in the basic cellular processes, were used as internal controls without proper validation of their presumed stability of expression [16]. However, the stability of common HKGs (e.g. *ACT, GAPDH* and *18S rRNA*) have been reported to vary considerably in given tissues or experimental conditions and therefore they are not generally suitable for all gene expression studies [17]. Generally, selecting an appropriate reference gene is done in two steps: (1) identification of the candidate reference genes, and (2) determination of their expression stability on representative samples. Meanwhile, different strategies such as NormFinder, geNorm and BestKeeper were developed to select the best HKGs based on model-based variance estimation approach [13], geometric averaging of multiple internal control genes [10] and pair-wise correlations [18], respectively.

In the most RNA preparation methods, a low level of genomic DNA (gDNA) usually remain in RNA samples that cause to non-specific amplification in qPCR [17] and consequently cause an overestimation of the transcription level. Caldana et al. [19] proposed the use of the intergenic regions and intron of the genes to monitoring of the gDNA contamination. Because of different accessibility of chromosomal sites to *DNase* I, it is emphasized to use different primer pairs that are spread on several chromosomes. In this study, the universal primers based on the ribosomal DNA (rDNA) were designed for the assessment of DNA contamination. In order to identify the most stably expressed reference genes, the expression of ten candidate reference genes participating in different biological processes was analyzed in two different tissues of *A. littoralis* at salt stress and recovery condition.

## Results

### DNA contamination assay

**Fig. 1:**
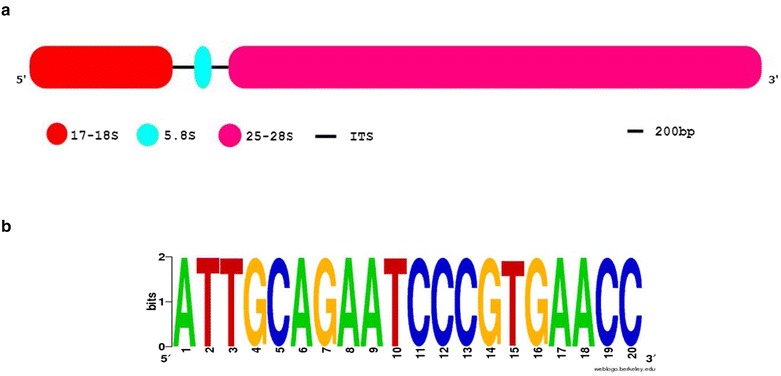
The rDNA structure and its conserved sequences. **a** Schematic representation of the eukaryotic rDNA segment that contains 17–18S, 5.8S, and 25–28S rRNA. **b** The motif logo of the 5.8S primer homology in 2000 green plants records (NCBI taxid number: 33090) by BLASTN with a cut-off (e-value ≤ 1 × 10^−10^)

The RNA samples have been tested by qPCR with three rDNA-based primer pairs. The rDNA genes generally consist the two internal transcribed spacer (ITS) regions including ITS1 and ITS2, and also three rRNA genes containing small subunit (17–18S or SSU), 5.8S and large subunit (25–28S or LSU) [20]. The schematic structure of eukaryotic rDNA is represented in Fig. 1a. Because the SSU and LSU rDNAs are highly conserved, the SF and LR primers were designed based on these regions (Table 1). The SSU-5.8S amplicon amplified by SF (forward) and R (reverse) primers is spanning the partial region of 17–18S rDNA, complete region of ITS1 and partial region of 5.8S rDNA. In the 5.8S-LSU amplicon, the partial region of 5.8 S rDNA, complete region of ITS2 and partial region of 25–28S rDNA was amplified by F and LR as forward and reverse primer, respectively. These primers can be used as universal qPCR primers in gDNA contamination assay especially for most of Poaceae species. The motif logo generated from 2000 green plant records (NCBI taxid number: 33090) for 5.8S primer binding site is shown in Fig. 1b. To test the universality and specificity of SF, LR and 5.8S primers and specificity of ITS1 primers, gDNA of several plant species including wheat (*Triticum aestivum*), barley (*Hordeum vulgare*), rice (*Oryza sativa*), alfalfa (*Medicago sativa*), *Aeluropus littoralis*, cucumber (*Cucumis sativus*), tobacco (*Nicotiana tabacum*), berseem clover (*Trifolium alexandrinum*), faba bean (*Vicia faba*) and *Arabidopsis thaliana* were analyzed by qPCR. The qPCR melt curve analysis indicated that the amplicons of SSU-5.8S and 5.8S-LSU had single peaks with no primer-dimer formation in all studied species (Additional file 1: Figure S1). Size separation of the PCR products showed a single band, however as expected the bands associated with different species had different size (Additional files 2: Figure S2A and S2B). It is interesting to note that application of these universal primers is not only restricted to the Poaceae species, but they also can be applied for analysis in other plant species and endophytic fungus e.g. *Piriformospora indica.* The ITS1 primer that it was designed based on *Aeluropus* species produced a single sharp band not only in *Aeluropus littoralis* gDNA but also have been observed in other species (with the exception of *Nicotiana tabacum* and *Trifolium alexandrinum*, where two bands were produced—Additional file 2: Figure S2C). Due to the high rate of mutation in ITS regions, the primers designed based on this regions generally are species-specific and maybe will not work in other species. Generally, the use of this ITS primer is proposed when the interaction of some species (plant–microbe interaction) is investigated together.

**Table 1:** Primer sequences, amplicon characteristics and expected amplicon size in DNA contamination assay

Amplicon (primer pair) name	Amplifying region	Sequence	Expected amplicon size (bp)
SSU-5.8S	Amplify partial 17–18S rRNA, complete ITS1 and partial 5.8 S rRNA	SF^†^: cgtaacaaggtttccgtaggtg R*: ggttcacgggattctgcaat	332–405
5.8S-LSU	Amplify partial 5.8 S rRNA complete ITS2 and partial 25–28S rRNA,	F*: attgcagaatcccgtgaacc LR^†^: tgcttaaactcagcgggtag	318–361
ITS1	Contains internal transcribed spacer 1	ggtatggcgtcaaggaacact atagcatcgctgcaagaggt	100–200

*SSU* small subunit; *LSU* large subunit; *ITS1* Internal transcribed spacer 1

*, ^†^ Denote primer universality in flowering and monocote plant species, respectively

The two regions of ITS1 and ITS2 are not part of the ribosome genes and are spliced and removed in mature rRNA. In our hypothesis, no amplification must be observed in *DNase*-treated RNA samples when two regions of SSU-5.8S and 5.8S-LSU are amplified. To monitor residual gDNA contamination in RNA sample, the total RNA samples were examined by these primers in qPCR. In this view, observation of any band on the agarose gel or melting curve peak in qPCR analysis could be considered as gDNA contamination. In this study, all RNA samples were tested by this procedure for DNA contamination assay.

### Selection of candidate reference genes

The 646 ESTs of *A. littoralis* were retrieved through Entrez Gene -EST database- at the National Centre for Biotechnology Information (NCBI). The candidate reference genes were chosen based on homology sequence analysis of ESTs using the BLASTN, tBLASTX and BLASTX algorithms. Ten reference genes from different biological processes and pathways were selected and are listed in Table 2. The gene name and symbol were presented base on gene ontology and the Arabidopsis Information Resource (TAIR, https://www.arabidopsis.org) annotations.

**Table 2:** Candidate reference genes were used for the assessment of the expression stability in qPCR analysis

Gene symbol	Accession no.	Name	GO annotation	e-value	Primer sequencec	Amlicon size
*ACT11*	EE594539.1	Actin-11	Structural constituent of cytoskeleton	1 × 10^−66^	GTATGGCAACATTGTCCTCAG TGGAGCAACGACCTTGAT	118
*U2SURP*	EE594692.1	U2snRNP-associated SURP motif-containing protein-like	RNA binding, required for spliceosome assembly to participate in splicing	3 × 10^−55^	CGTGGATGAGATTGAGAGGAA TGGAGGACTACGGCTTCTA	199
*EF1A*	EE594715.1	Elongation factor-1 alpha	Translation elongation factor activity	1 × 10^−04^	TGCTGTCGGTGTCATCAA CTTCCATCAAACGCCTCATT	97
*UBQ*	EE594598.1	Ubiquitin-like protein	Biologically significant role in protein delivery to proteasomes and recruitment of proteasomes to transcription sites.	9 × 10^−19^	CTTGGTCTGCTGTTGTCTTG CACGGTTCACTTATCCATCAC	200
*TUB*	EE594551.1	Beta-tubulin chain-like	Microtubule-based process and structural constituent of cytoskeleton	5 × 10^−20^	TGCTGCCTGCTGTATCTT CGGAGGAACTTACTACTACATACT	109
*eIF3*	Jk671263	Eukaryotic translation initiation factor 3 subunit B-like	Translation initiation factor activity	1 × 10^−90^	CCGCCATCGCTACTGTCTCC CCTCTTGCGCTCCTGTTCACT	126
*GTF*	JZ191082.1	General transcription factor 3C polypeptide	Involved in RNA polymerase III-mediated transcription	8 × 10^−39^	TTCCAAGTGGCCATCAGGTT AAAGGGCTTCCTGCCTCTTG	108
*RPS12*	JZ191056.1	40S ribosomal protein S12-like	Structural constituent of ribosome involved in RNA methylation, photorespiration, translation	1 × 10^−91^	TTGGCAGACTCACGAAGG GATGGCGGATCAGGAGAC	147
*GAPDH*	JN604531.1	glyceraldehyde-3-phosphate dehydrogenase	Dehydrogenase, Oxidoreductase in glycolysis and gluconeogenesis	0.0	TGGGCAAGATTAAGATCGGAAT TTGATGTCGCTGTGCTTCCA	184
*RPS3*	JZ191044.1	40S ribosomal protein S3-like	Structural constituent of ribosome involved in RNA methylation, photorespiration, translation	2 × 10^−59^	ATTCACTGGCTGACCGGATG GTGCCAAGGGTTGTGAGGTC	107

Gene names are denoted base on gene ontology and TAIR annotations

### Primer and reaction validation

**Fig. 2:**
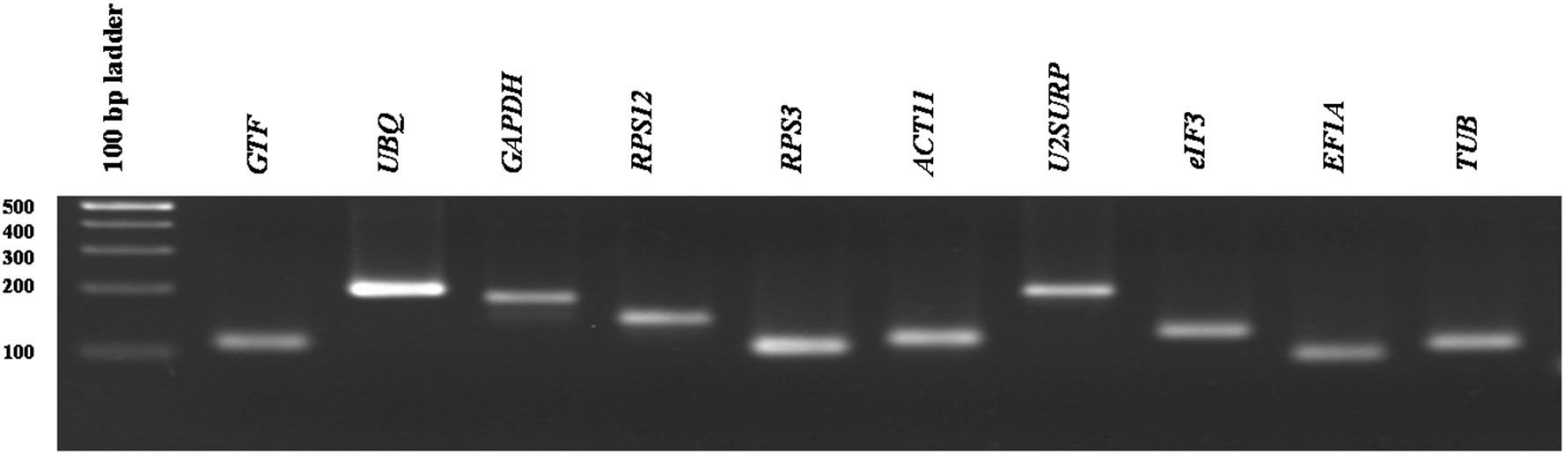
The amplicon of 10 candidate reference genes was analyzed on 3 % agarose gel

The cDNA synthesized from control and treatment samples from root and leaf tissue was analyzed by qPCR (N = 8). Pooled cDNA tissue samples containing equal amounts of the cDNA from control and all treatments were used to determine the primer pairs annealing temperature and their specificity. Primers annealing temperature was adjusted to 60 °C, and their specificity was checked by melt curve analysis. Single sharp peak with no primer-dimer was observed in all primers. The melt curve analyses of the amplicons associated with root samples are given in Additional file 3: Figure S3. Agarose gel electrophoresis in 3 % gels also confirmed the size of the amplicons associated with reference genes (Fig. 2). The fivefold serial dilution with the same pooled cDNA was used for sample and primer validation. The calibration curve was calculated from the results of a set of serially-diluted cDNA samples (Additional file 4: Figure S4). Results showed that the PCR efficiency in root and leaf samples ranged from 72.4 to 102.8 % and 84.8 to 115.9 %, respectively. The R^2^ of primers were greater than 0.986 (Additional file 5: Table S1). Also, the single curve efficiencies determined by LinRegPCR program [21] were used to estimate the PCR efficiency for each individual sample and reaction. The LinRegPCR analysis indicated that all reactions had e-values greater than 82 % by R^2^ ≥ 0.998. In this study, efficiencies derived by LinRegPCR do not correspond to efficiencies calculated by standard curve analysis. In a number of published manuscripts, the mean of the single curve-derived efficiencies gave less biased results than a standard curve-derived efficiency [22]. Because of standard curve-derived efficiency does not show the true mean PCR efficiency of the samples, mean efficiencies per amplicon derived by LinRegPCR were used in all gene expression calculations.

### Cq values analysis

**Fig. 3:**
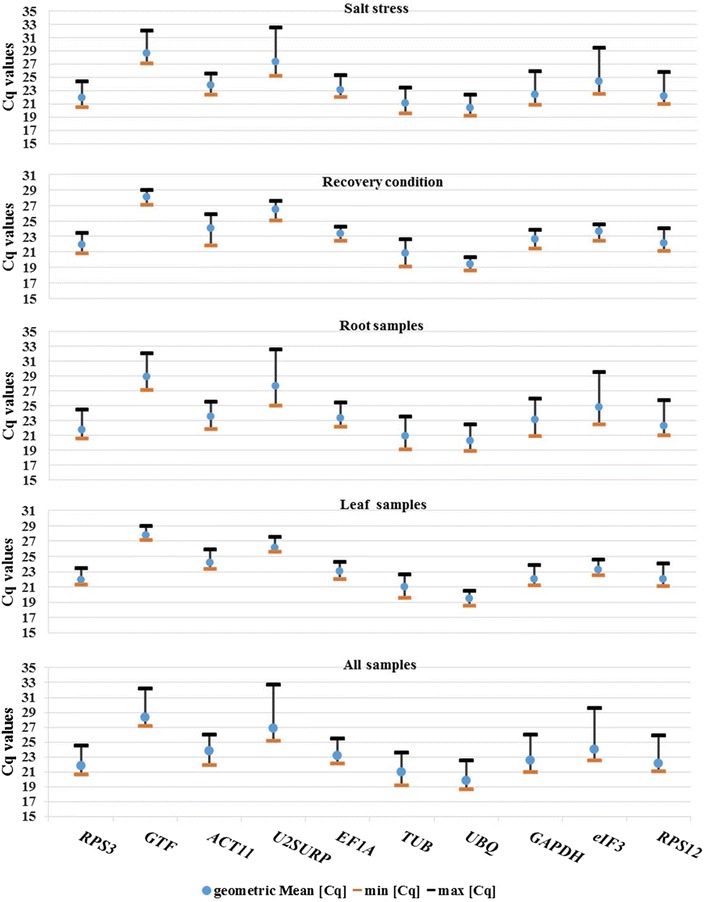
*Box-plot* of Cq values for the 10 candidate reference genes among all cDNA samples. The median values are showed as a *line* across the *box*. The first and third quartiles are represented as *red* and *yellow boxes*, respectively. The *lower* and *upper whiskers* indicate the highest and lowest values, respectively

All of the qPCR data obtained from ten candidate reference genes (N = 16) were used for quantification cycle (Cq) value analysis by BestKeeper program (Additional file 6: Table S2E). In this study the mean Cq values of the ten specific PCR reactions ranged from 19.86 for *UBQ* to 26.85 for *U2SURP*. *UBQ* and *TUB* showed relatively high level of mRNA with mean Cq of 19.86 and 20.94, respectively. *GTF* and *U2SURP* displayed the lower amount of mRNA with mean Cq of 28.36 and 26.85, respectively. The mean Cq of other candidate reference gene ranged from 21.86 to 24.01 with moderately abundant mRNA level. To estimate the difference between each Cq value from the mean, standard deviation (SD) of the Cq value were determined across root (N = 8), leaf (N = 8), salt stress (N = 8), recovery condition (N = 6) and over all samples (N = 16). Each set of root and leaf samples, was consisting of four time-course of salt-stressed plants (6, 24, 48 h and 1 week) and three time-course of recovered plants (6 , 24 h and 1 week) as well as control plants. The four time-course of root and leaf stressed plants (6, 24, 48 h and 1 week) was considered as salt stress treatments (N = 8) while the three time-course of root and leaf recovered plants (6, 24 h and 1 week) were evaluated in recovery condition analysis (N = 6). Among the all candidate reference genes, the expression level of *U2SURP* and *eIF3* showed the highest Cq variability with SD of 1.58 and 1.37, while the mRNA amount of *UBQ* and *RPS3* displayed the lowest Cq variability with SD of 0.75 and 0.80, respectively (Fig. 3). It was stated that each value with the SD higher than 1 could be considered as inconsistent genes with low expression stability [18]. The mRNA expression of all candidate reference genes in the leaf and recovered plants had low variability with SD values lower than one except for *ACT11* in recovered plants with the value of 1.27 (Fig. 3 and Additional file 6: Table S2).

In this study, the Cq variation of candidate reference genes in the leaf and recovered plants were low as expected. It should be noted that during recovery treatment the plant returns to normal condition (partially or completely) and enable damage repair upon stress relief. *GTF* (0.39) and *U2SURP* (0.48) had lowest SD value of expression level in the leaf while *UBQ* (0.51) and *eIF3* (0.53) had lowest SD value in recovered plants. As represented in Fig. 3, the root and salt stressed samples had a high Cq variation and SD value. The expression level of *UBQ*, *RPS3* and *EF1A* in the roots and the expression level of *ACT11*, *UBQ*, *TUB* and *RPS3* in the salt stressed plants had a SD value lower than one. The high Cq variation in the root samples may be related to direct contact of root cells with salt stress. As the roots are the first line of defense when the cells encounter salinity stress, the diversity in their gene expression is expected.

### Expression stability analysis

In order to select the best candidate reference gene, the qPCR results were analyzed by BestKeeper, geNorm and NormFinder programs. In analysis of expression stability of HKGs by BestKeeper program, the descriptive statistics of each HKG were computed. The descriptive statistics of the derived quantification cycle (Cq) of each HKG are given in Additional file 6: Table S2. BestKeeper determined the optimal HKG by applying the pair-wise correlation analysis of all pairs of candidate genes, and select the best ones based on variables of SD, percent covariance and power of the reference gene [17, 18]. The integrity of samples (uniformity in quantity and quality of starting mRNA and cDNA preparations) as well as their expression stability was checked by an intrinsic variance (InVar) factor (Additional file 7: Table S3). Based on InVar factor, the most of samples had low Cq variation with the few fold changes in expression level. Two of the root investigated samples had higher variation in Cq value and were excluded from BestKeeper analysis (removal often recommended over the threefold changes). Our analysis showed that the most Cq SD values in all remained-samples (N = 14) varied from 0.47 to 0.81 cycle. Analysis of the pair HKG relations of all possible combination across all remained-samples (N = 14) showed a strong correlation (0.324 ≤ r ≤ 0.906). Although the high Cq variation (SD value above one) were observed in expression level of *U2SURP*, *GAPDH*, *eIF3* and *GTF*, but after removing two root samples, the Cq variation of all candidate reference genes has been become lower than one. The rankings of candidate reference genes derived from BestKeeper expression stability analysis are given in Additional file 9: Table S5.

The *RPS3*, *UBQ*, *RPS12*, *eIF3* and *EF1A* had high degree of expression stability in root samples while in leaf samples ranking of top five HKGs were as follows: *GTF* > *U2SURP* > *UBQ* > *eIF3* > *EF1A*. When all samples were analyzed together in BestKeeper, the top five HKGs ranking were as follow: *UBQ* > *GTF* > *eIF3* > *RPS3* > *RPS12*/*EF1A*. The five candidate genes of *ACT11* > *UBQ* > *TUB* > *RPS3* > *EF1A* had highest expression stability across salt-stressed plants, while the order of top most stably expressed gene in recovery condition was as follows: *UBQ* > *eIF3* > *EF1A* > *GAPDH* > *GTF*.

**Fig. 4:**
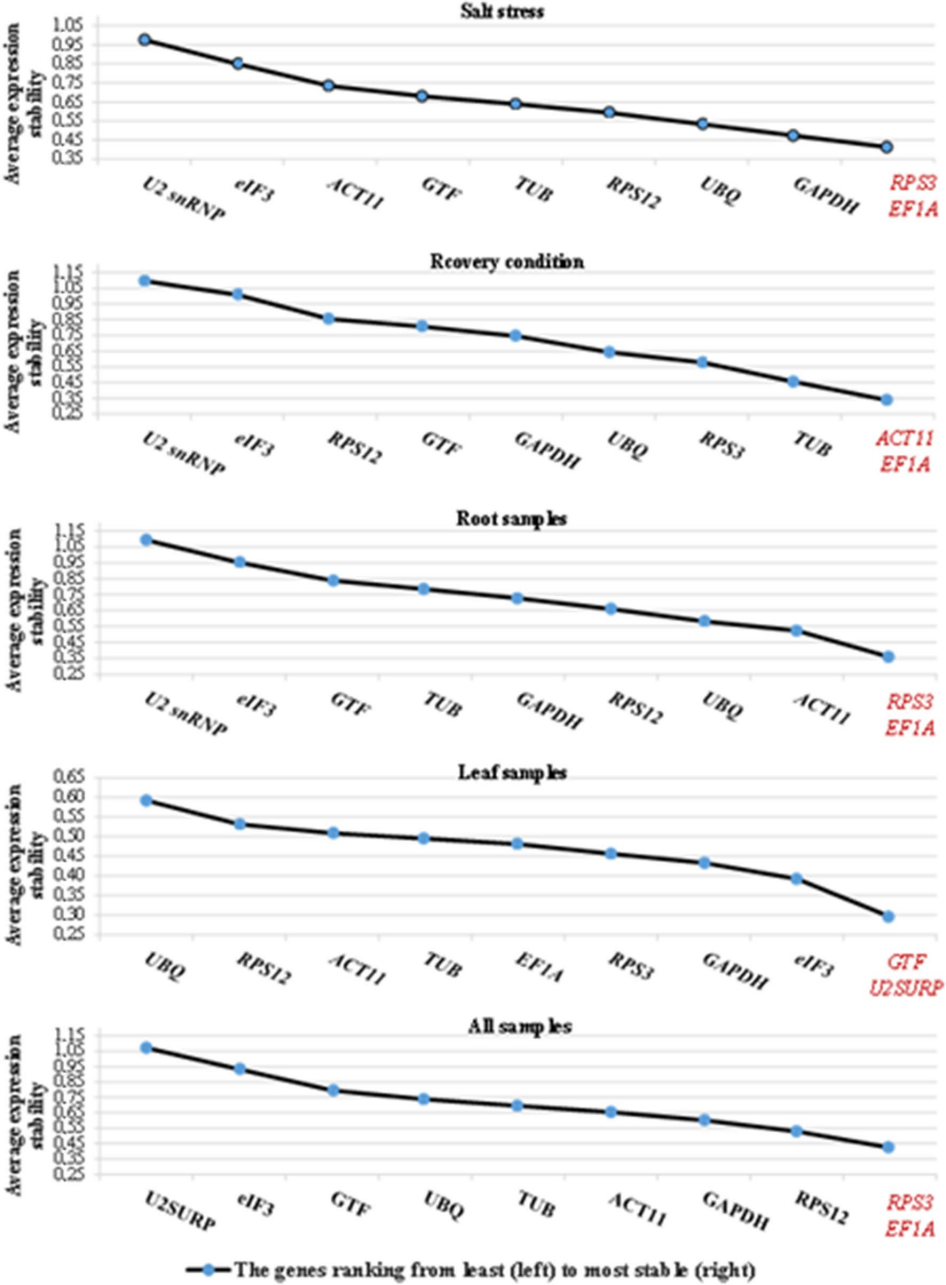
The average expression stability of 10 candidate reference genes in salt stress, recovery condition, root, leaf and all 16 cDNA samples by geNorm program. The two most stably expressed genes are represented by *red color*

**Fig. 5:**
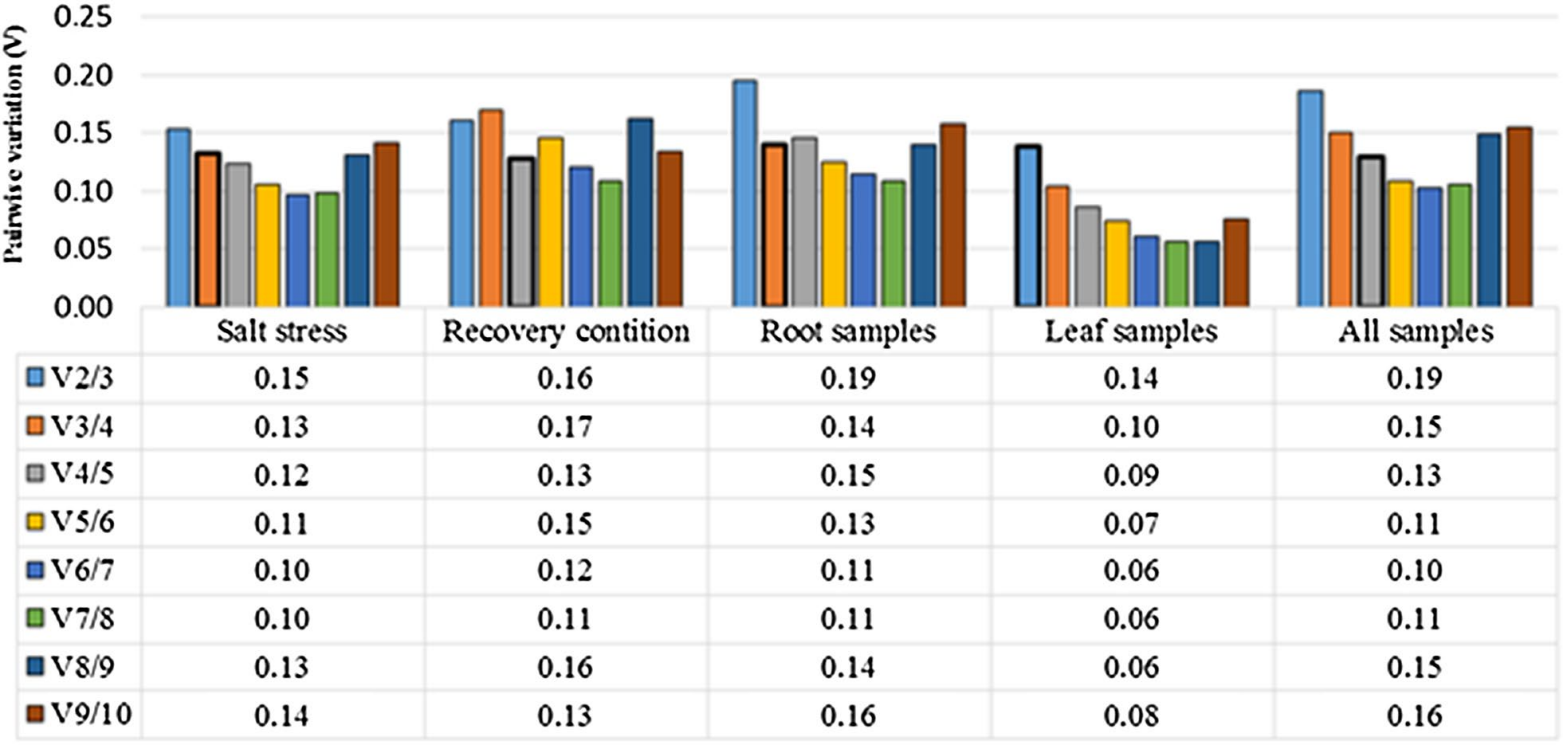
Determination of the optimal number of reference genes by pairwise variation (V) analysis in geNorm program at different tissues and conditions. The optimal reference genes number according to their V value (lower than 0.15) is signed

The average expression stability values (M) in the geNorm program were calculated based on the average pairwise variation (V) for one reference gene with all other tested reference genes. Stepwise exclusion of the gene with the highest M value allows ranking of the tested genes according to their expression stability [10]. In this study, the transformed Cq values to linear expression quantities were used in geNorm analysis. The two genes of *RPS3* and *EF1A* were identified as the most stably expressed genes in salt-stressed and recovered plants as well as all samples (Additional file 8: Table S4A and Fig. 4). In the recovered plants, *ACT11* and *EF1A* were ranked as the most stably expressed while the *RPS3* occupied the fourth rank. Expression analysis of the leaf indicated that *GTF* and *U2SURP* genes were more stably expressed followed by *eIF3*, *GAPDH*, *RPS3* and *EF1A* genes. The order of expression stability of the 10 reference genes are represented in Additional file 9: Table S5. The three genes of *U2SURP*, *eIF3* and *GTF* showed the lowest expression stability among all grouped samples except for the leaf tissue. The analysis of root samples indicated that *RPS3*, *EF1A* and *ACT11* were more stably expressed, while *U2snRNP* and *eIF* showed least expression stability. An optimal number of reference genes for accurate normalization was determined by pairwise variation (V) analysis among candidate reference genes (Fig. 5). The cut-off value—below that one the inclusion of an additional reference genes for the normalization factor is not required—was set at 0.15 (V < 0.15). In the root, V3/4 value was 0.141, and therefore at least a set of three most stably expressed gene including *RPS3*, *EF1A* and *ACT11* were selected. In the leaf the V2/3 value was 0.13, and at least the two best reference genes of *GTF*/*U2SURP* were proposed for normalization. For relative comparison of all 16 samples, preferably the four best reference genes including *RPS3*, *EF1A*, *RPS12* and *GAPDH* were chosen (V4/5 = 0.12). In salt-stressed plants (V3/4 = 0.13), three genes of *RPS3*, *EF1A* and *GAPDH* were selected while and in recovered plants (V4/5 = 0.12), the four genes of *ACT11*, *EF1A*, *TUB* and *RPS3* were proposed.

In contrast to geNorm program, the NormFinder, by calculating expression stability of each single gene independently, shows less sensitivity to co-regulation of the reference genes [13, 17]. In NormFinder, those genes with the lowest stability value were supposed to have the highest expression stability. The results of NormFinder based on different tissues and conditions are given in Additional file 8: Table S4B. *EF1A* and *eIF3* with the stability value of 0.048 and 0.023 were selected as best reference genes in root and leaf, respectively. When all samples (N = 16) were considered together, *EF1A* with stability value of 0.038 were selected as the most stably expressed gene. Stability value for the best combination of two genes was 0.028 that belong to the *GTF* and *EF1A*. The top candidate genes in salt-stressed plants were observed as follows: *RPS3* > *RPS12* > *GTF* > *GAPDH* > *EF1A*. The order of most stably expressed gene in recovered plants was as follows: *EF1A* > *GTF* > *GAPDH* > *RPS3* > *ACT11* (Additional file 9: Table S5).

### Appropriate reference genes

The order of ten candidate reference genes based on BestKeeper, geNorm and NormFinder programs is given in Additional file 9: Table S5. According to the results of three different statistical approaches, the candidate reference genes were not ranked in the same order. The genes ranking in geNorm and NormFinder under salt stress conditions were more consistent with each other compared to the BestKeeper results. In contrast, at recovery condition, the ranking generated by BestKeeper and geNorm was in better accordance with each other. It has been previously shown that applying different software for normalization using distinct statistical algorithms and analytical procedures sometimes produce different results [23, 24]. Based on the geNorm analysis, the optimal number of reference genes for each case of salt, recovery, root, leaf and all selected samples was suggested to be at least three, four, three, two and four genes, respectively. Regarding to these optimal number of reference genes and comparing the results of the three applied methods, five different set of most stably expressed genes were proposed for each condition and tissue (Table 3). The three genes (namely, *RPS3*, *EF1A* and *UBQ*) were recommended as normalizer in salt-stressed samples and root. The genes *RPS3*, *EF1A*, *UBQ* and *GAPDH* were selected as reference genes for recovered plants while two HKGs (*U2SURP* and *GTF*) were chosen for leaf. Finally, for relative gene expression analysis of all samples, the set of four reference genes including *RPS3*, *EF1A*, *GTF* and *RPS12* is recommended.

**Table 3:** A set of optimal reference genes is recommended for each sample group

Sample groups	Optimal reference genes
Salt stress	*RPS3, EF1A* and *UBQ*
Recovery condition	*RPS3, EF1A,UBQ* and *GAPDH*
Root	*RPS3, EF1A* and *UBQ*
Leaf	*U2SURP* and *GTF*
All selected conditions and tissues	*RPS3, EF1A, GTF* and *RPS12*

## Discussion

The qPCR approach is one of the most widely used techniques for gene expression analysis. The advantage of this method is the sensitivity, specificity, accuracy and reproducibility. However, to get reliable results especially when different tissues and treatments are examined, the accurate normalization of gene expression against a reference genes is a critical prerequisite [10]. On the other hand, the high specificity of primers is required in qPCR to prevent any non-specific amplification that arises from primer dimer or gDNA. Quality control (QC) of RNA preparation particularly in terms of gDNA contamination is important for the correct interpretation of the data obtained from qPCR. It is expected that contamination of RNA samples with gDNA will lead to an overestimation of the RNA transcript level [17]. No reverse-transcriptase (NRT) control typically is included in qPCR experiments for evaluation of the amount of gDNA contamination that present in RNA samples. In this study, we proposed a qPCR-based approach for monitoring gDNA contamination that eliminates the need for NRT in each reaction. The two unique universal primer pairs based on rDNA genes including SSU-5.8S and 5.8-LSU were proposed for tracking of DNA contamination in Poaceae. The eukaryotic genomes contain up to thousands of rDNA genes that can be arranged in tandem repeating units (placed side by side) [25]. The main role of these genes is the generation of ribosomal subunits required for protein synthesis. Transcription of these units is initialized by RNA polymerase I (RNA pol I) in a polycistronic manner. The rDNA genes are initially transcribed to a pre-rRNA, which is then processed by removing of the two internal transcribed spacer regions (including ITS1 and ITS2) to produce the three mature rRNAs including 17–18S rRNA, 5.8S and 25–28S rRNA [20]. Owing to the nature of multigene family, highly conserved sequences, high copy numbers in the genome and potential presence at more than one chromosomal location [26], the rDNA genes compared to intronic sequences of protein coding genes represent good DNA regions for reliable tracking of DNA contamination in the most flowering plants. However, the use of ribosomal RNA as proper reference genes in relative gene expression analysis is problematic due to several disadvantages, such as different biogenesis, imbalance between rRNA and mRNA fractions, use of hexamer primers and overestimation in mRNA copy numbers (up to 19-fold) [19, 27]. But some aforesaid constrains could be considered an important advantage for DNA contamination monitoring. For instance, high frequency of these multigene rDNAs significantly increased the capability of DNA contamination detection and then improved the sensitivity. These unique universal primer pairs probably amplify the same regions on the different chromosomes and thus could monitor the presence of the residual gDNA contamination based on various chromosomal regions. It should be noted that the universality and specificity of these primer pairs in different plant species were also addressed in this study (Additional files 1: Figure S1 and S2). Because of the high rate of mutation in these ITSs, they are widely used for studying phylogenetic relationship at the inter- and intra-specific levels in plants [26]. In gene expression analysis, the species-specific primers based on these regions not only can be used for gDNA contamination assay, but also could be especially applied in absolute quantification analysis at plant–microbe interaction studies.

It is likely that the transcriptome of each cell type is very different from that of other cell types, tissues and organs [28] or experimental conditions. Results of many reports suggest that the HKGs are regulated differently in different plant species [29]. Meanwhile, the best reference genes in one organism cannot be generalized to another organism at a given experiment [16, 19, 24]. It should be noted that the use of single HKGs, typically ACTB or GAPDH is a common strategy for normalization in most the qPCR relative analysis. However, it has been estimated that the magnitude of change of these genes varies up to 10-fold across different samples [17]. Because of the expression variability of HKGs in different tissues and conditions, it is proposed that more than one reference gene should be used for qPCR normalization [10, 13, 17]. To identify the most stably expressed reference genes in *A. littoralis* we initially selected ten candidate references genes, some of them (such as EF1A, GAPDH and UBQ, eIF and ACTB) are commonly used as HKGs in plants. The expression stability of these genes was analyzed across the root and leaf tissue and salt-stress and recovered plants. Among these most common HKGs reported in many studies, EF1A, GAPDH and UBQ displayed the highest expression stability in this study. In contrast, the expression of eIF3 and ACT11 genes among candidate reference genes showed high expression variation in some examined tissues and conditions. Because of the wide HKGs expression variation across samples, we recommend using multiple reference genes for each condition and tissue.

By all applied methods (BestKeeper, geNorm and NormFinder), a set of three reference genes including *RPS3*, *EF1A* and *UBQ* had highest rank in comparisons, and therefore their Cq geometric mean could be used as normalizer at salt-stressed, root and recovered samples in qPCR expression analysis. Here we propose novel reference gene e.g. *RPS3* with a much lower level of variance in expression across tissues and experimental conditions than commonly used housekeeping genes. *RPS3* encodes ribosomal proteins involved in protein biosynthesis. Also, the *U2SURP* and *GTF* had highest expression stability in leaf. *U2SURP* as a functional spliceosome-associated protein and *GTF* as tightly associated component of the DNA-binding TFIIIC2 subcomplex involve in RNA processing, and RNA polymerase III-mediated transcription, respectively.

## Conclusion

In conclusion, the use of two unique rDNA-based primer pairs (e.g. SSU-5.8S and 5.8S-LSU) was addressed for tracking residual DNA in RNA samples. These primers act as intron-flanking primers, and by their multiple targeting site on the different chromosomes are more sensitive in comparison to other common intron-based primers. Also by applying these primers, the need for NRT control could be eliminated in each qPCR experiment. Since the genomic annotation of the most wild plants is not available by now, the qPCR primer design based on exon–exon expanding is not possible yet. But, according to specificity, universality and versatility of these proposed primer pairs, the gDNA contamination detection of the Poaceae and the most flowering plant species is achievable. To our knowledge, this is the first report that explains application of these unique universal primer pairs in gene expression and qPCR analysis. Also, this is the first study to validate a set of candidate reference genes for normalization of expression levels in *A. littoralis* as a representative of halophytic Poaceae. We provided five set of reference genes as well as their optimal number by using the three programs BestKeeper, geNorm and NormFinder for each tissue and condition, i.e., root, leaf, salt-stressed and recovered plants. Comparing the results of three algorithms showed that the two genes of *EF1A* and *RPS3* had high rank in all given experiments except leaf, and were proposed as common reference gene in our study. Here we showed novel candidate reference genes e.g. *RPS3* with much higher expression stability among different tissues and experimental conditions than commonly used housekeeping genes. Our identified candidate reference genes can be used in future qPCR experiments on *Aeluropus littoralis* studies.

## Methods

### Plant material

*Aeluropus littoralis* seeds were collected from Isfahan province (Roddasht region) in Iran and the sterilized seeds plated on full strength MS medium with vitamins, 3 % sucrose and 0.7 % agar (pH 5.8). Two weeks after germination, the seedlings were transferred to hydroponic culture containing Hoagland’s solution. The 30 day-old seedlings were stressed in 600 mM of sodium chloride at six passages (received 100 mM sodium chloride per 48 h up to 600 mM). Leaves and roots were sampled in parallel. At the end of the sixth passage, samples were collected at 6, 24, 48 h and 1 week time-course. In order to plant recovery, the remained plants were transferred to a sodium chloride-free Hoagland’s solution, and then were collected after 6, 24 h and 1 week. All samples as well as control were immediately frozen in liquid nitrogen and stored at −70 °C for RNA extraction.

### Total RNA and DNA extraction

Total RNA was extracted using TRIzol reagent (Invitrogen Life Technologies, Karlsruhe, Germany) according to the manufacturer’s instructions. For the test of universality of rDNA-based primers, DNA of several plant species including wheat (*Triticum aestivum*), barley (*Hordeum vulgare*), rice (*Oryza sativa*), alfalfa (*Medicago sativa*), *Aeluropus littoralis*, cucumber (*Cucumis sativus*), tobacco (*Nicotiana tabacum*), berseem clover (*Trifolium alexandrinum*), faba bean (*Vicia faba*) and *Arabidopsis thaliana* as well as the endophytic fungus *Piriformospora indica* was extracted according to Dellaporta procedures [30]. The quality and quantity of the extracted nucleic acid were checked by measuring absorbance at 260/280 nm using a NanoDrop spectrophotometer (Biochrom WPA Biowave II, UK). Further, the purity and integrity of RNA and DNA were tested by running on 1.2 and 0.7 % agarose gel electrophoresis, respectively.

### DNA contamination assay

Residual gDNA contaminating RNA extracts was removed by DNase treatment (*DNase* I RNase-free, Thermo Scientific, USA). The qPCR with three rDNA-based primers was applied for DNA contamination assay by using RNA as template. The forward and reverse primer on the 5.8 S rRNA target were designed based on a conserved motif [31] in the 5.8S ribosomal RNA gene in flowering plants. The 17–18S forward primer and 25–28S reverse primer were designed based on conserved regions in Poaceae. The ITS primer sequences were designed based on conserved regions in ITS1 of *Aeluropus* (NCBI taxid number: 110873). BLAST searches were carried out using public database at NCBI. The conserved regions from various species were aligned by ClustalW [32]. rDNA-based primers were designed after taxa specific/cross-species analysis with AlleleID v7.0 software (Premier Biosoft International, Palo Alto, CA). Primers were selected according their specificity and universality by BLASTN search. Motif logo of 5.8S primer was generated by WebLogo (http://weblogo.berkeley.edu). The primer sequences are presented in Table 1.

### Bioinformatic analysis, reference gene primer design and validation

The EST sequences of *Aeluropus littoralis* were retrieved from EST database at NCBI and were analyzed using the BLASTN, tBLASTX and BLASTX algorithms [32]. The database of gene ontology (http.//http://www.geneontology.org) was used to investigate the molecular function of each EST and its role in biological processes as well as its location in the cell. After selection of candidate reference genes, the gene-specific primers were designed using the Beacon designer 8.02 (Premier Biosoft International, Palo Alto, CA), and were synthesized by Metabion GmbH (Martinsried, Germany). All designed primers had 18–24 length, GC content ranging from 42 to 61 % and similar melting temperatures (63–64 °C). The amplicon length ranged from 97 to 199 bp. The primer sequences and GenBank accession numbers of related genes are presented in Table 2. The primer specificity was evaluated by melt curve analysis, and size of the amplicons was tested by end-point PCR on 3 % agarose gels. The single curve efficiencies by LinRegPCR program were used to estimate the PCR efficiency for each individual reaction. Also, a mix cDNA sample containing equal amounts of the reverse-transcribed RNA from all control and treatment samples was used to estimate the reaction efficiency. Serial dilutions (1:1, 1:5, 1:25, and 1:125) of the root and the leaf mix cDNA samples were run concurrently in triplicate for each primer pair. According to MIQE guidelines [14] standard curves were generated by plotting the log starting cDNA quantity input versus the cycle threshold values of each reaction to determine the slope values and correlation coefficients (R^2^). The primer efficiency (E) was calculated using the following equation:

$${\text{E}} = \left( { 10^{{ - 1/{\text{slope}}}} {-} 1} \right) \; \times \; 100$$

### cDNA synthesis and qPCR analysis

The cDNA was synthesized using the QuantiTect reverse transcription kit (Qiagen, Germany) according to the manufacturer’s instructions. The final cDNA reactions were diluted 1:10, and stored at −20 °C. Targets were amplified by the Maxima SYBR Green/ROX qPCR Master Mix (Thermo Scientific, USA) with two-step cycling in CFX96 real-time PCR instrument (Bio-Rad, USA) according to the company’s suggestions. After amplification, all PCR reactions were subjected to a thermal melt with continuous fluorescence measurement from 55 to 95 °C for dissociation curve analysis. Curves were analyzed by CFX Manager (Bio-Rad) with single threshold cycle and subtracted curve fit method. At least one non-template control (NTC) was used for each primer pair master mix. The amplification efficiency for each reaction was calculated by LinRegPCR [22]. For assessment of the expression stability of the candidate reference genes across samples of the root and leaf, salt stress and recovery the quantification cycle (Cq) values were analyzed using geNorm [10], NormFinder [13] and BestKeeper [18].

## Additional files

10.1186/s40709-016-0053-8 The melt curve analysis of rDNA-based primers including SSU5.8S, 5.8SLSU and ITS1 in different species.
10.1186/s40709-016-0053-8 Agarose gel analysis of rDNA-based PCR product.
10.1186/s40709-016-0053-8 Melt curve analysis of ten candidate reference genes in a root samples.
10.1186/s40709-016-0053-8 The standard curve analysis for calculation PCR efficiency in root (A) and leaf (B) samples.
10.1186/s40709-016-0053-8 The primer features of candidate reference genes and their PCR efficiencies.
10.1186/s40709-016-0053-8 Expression stability analysis by BestKeeper.
10.1186/s40709-016-0053-8 Analysis of sample integrity by BestKeeper.
10.1186/s40709-016-0053-8 Expression stability analysis by geNorm and NormFinder.
10.1186/s40709-016-0053-8 Ten candidate reference genes in order to higher to lower expression stability were ranked by different approaches.

## Abbreviations

qPCR: quantitative real-time polymerase chain reaction
HKG_s_: housekeeping genes
gDNA: genomic DNA
rDNA: ribosomal DNA
rRNA: ribosomal RNA
ITS: internal transcribed spacer
SSU rRNA: small subunit rRNA
LSU: large subunit rRNA
NRT: no reverse-transcriptase control
NCBI: the National Centre for Biotechnology Information
