# Supplementary material for: Identification and validation of Aeluropus littoralis reference genes for Quantitative Real-Time PCR Normalization
Source: J Biol Res (Thessalon). 2016 Jul 19;23:18. doi: 10.1186/s40709-016-0053-8 (PMC4950632; doi:10.1186/s40709-016-0053-8)

**Supplementary Figure 1.** The melt curve analysis of rDNA-based primers including SSU5.8S, 5.8SLSU and ITS1 in different species. The samples and NTCs are shown by different and black color, respectively.


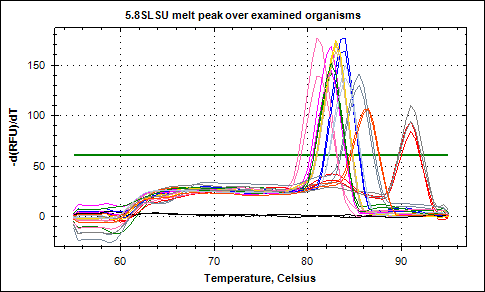


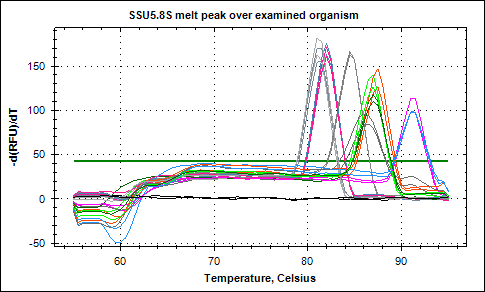


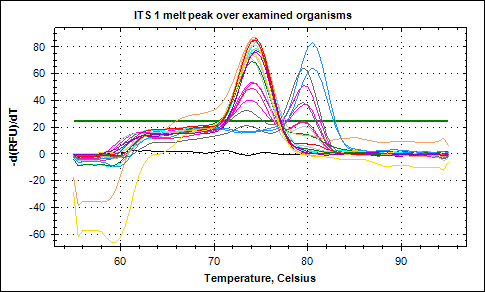

Supplement: Supplementary file 1 — 10.1186/s40709-016-0053-8 The melt curve analysis of rDNA-based primers including SSU5.8S, 5.8SLSU and ITS1 in different species. [file 40709_2016_53_MOESM1_ESM.docx]
