# Supplementary material for: Identification and validation of Aeluropus littoralis reference genes for Quantitative Real-Time PCR Normalization
Source: J Biol Res (Thessalon). 2016 Jul 19;23:18. doi: 10.1186/s40709-016-0053-8 (PMC4950632; doi:10.1186/s40709-016-0053-8)
Supplement: Supplementary file 2 — 10.1186/s40709-016-0053-8 Agarose gel analysis of rDNA-based PCR product. [file 40709_2016_53_MOESM2_ESM.docx]

**Supplementary Figure 2.** Agarose gel analysis of rDNA-based PCR product. The PCR product of SSU5.8S (A), 5.8SLSU (B) and ITS1 (C) were run on 3% agarose gel.

**100 bp Plus DNA Ladder**

***Triticum aestivum***

***Hordeum vulgare***

***Oryza sativa***

***Medicago sativa***

***Aeluropus littoralis***

***Trifolium alexandrinum***

***Arabidopsis thaliana***

***Nicotiana tabacum***

***Cucumis sativus***

***Vicia faba***

***Piriformospora indica***

**
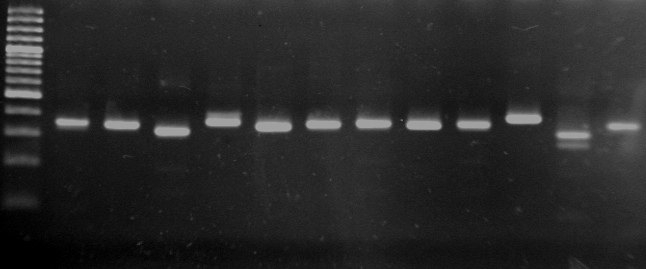
**
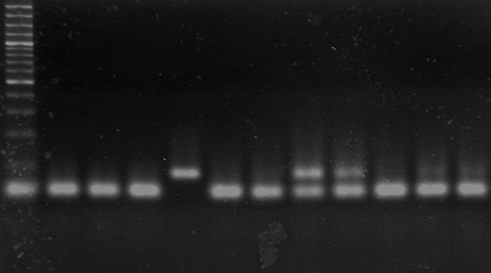
**
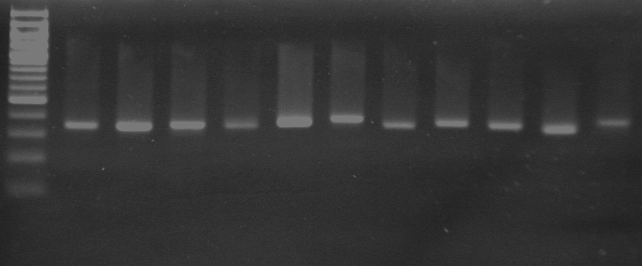
**

**B**

**C**

**A**

**3000**

**1000**

**500**

**400**

**300**

**200**

**100**

**3000**

**1000**

**500**

**400**

**300**

**200**

**100**

**3000**

**1000**

**500**

**400**

**300**

**200**

**100**
