# Supplementary material for: Identification and validation of Aeluropus littoralis reference genes for Quantitative Real-Time PCR Normalization
Source: J Biol Res (Thessalon). 2016 Jul 19;23:18. doi: 10.1186/s40709-016-0053-8 (PMC4950632; doi:10.1186/s40709-016-0053-8)
Supplement: Supplementary file 3 — 10.1186/s40709-016-0053-8 Melt curve analysis of ten candidate reference genes in a root samples. [file 40709_2016_53_MOESM3_ESM.docx]

**Supplementary Figure 3.** Melt curve analysis of ten candidate reference genes in a root samples. The samples and their NTCs are shown by green and blue color, respectively.


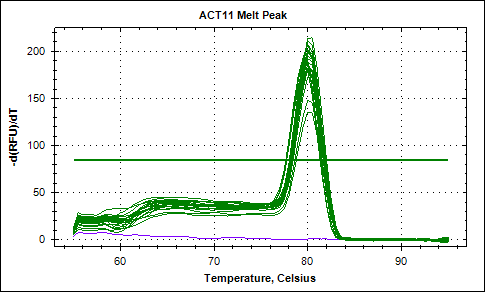

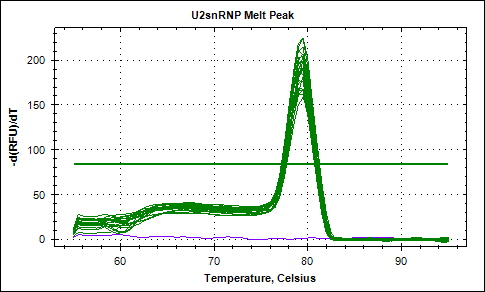


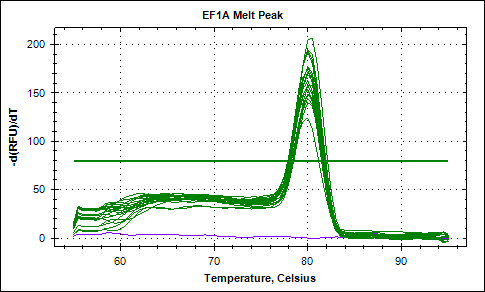

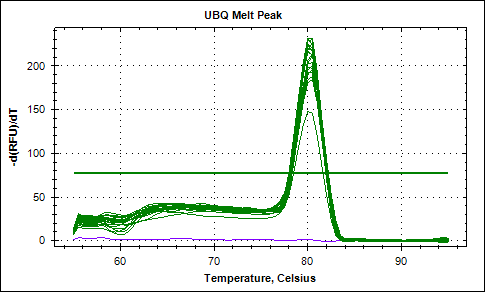


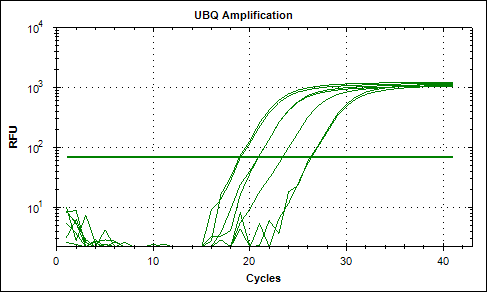

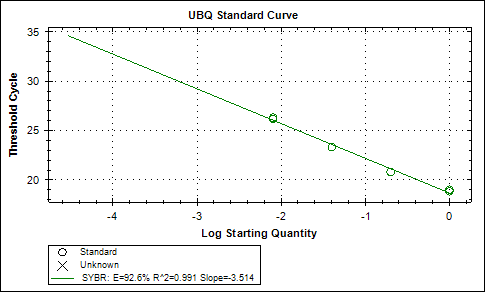


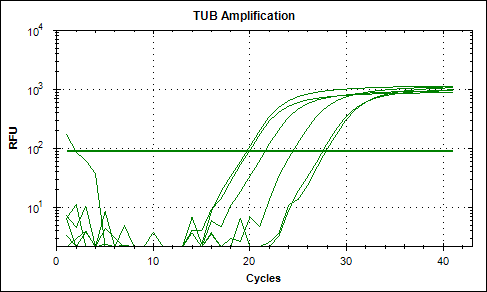

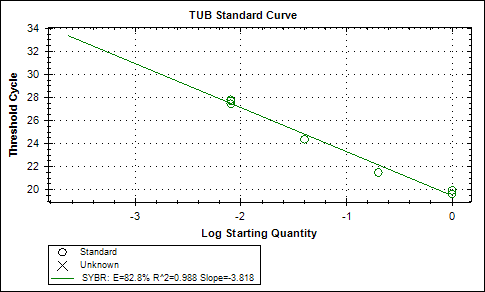


**U2SURP Melt pick**


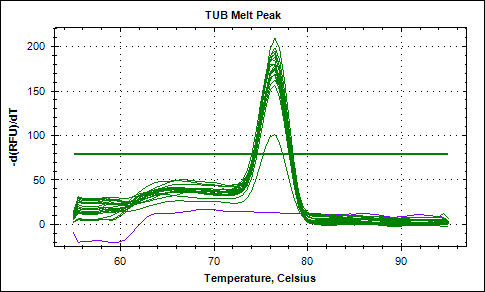
 **
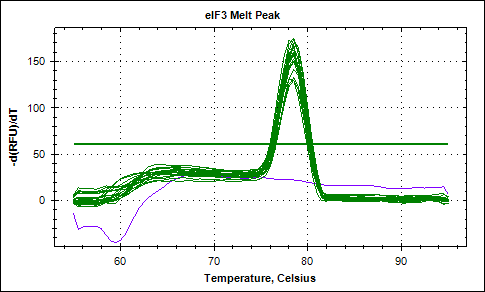
**


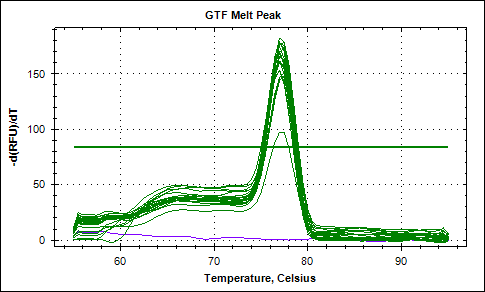


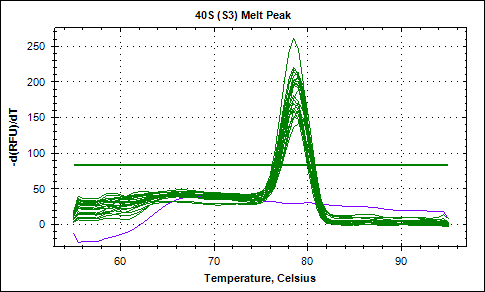


**RSP3 Melt Pick**


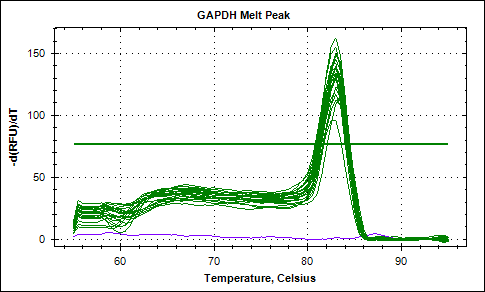


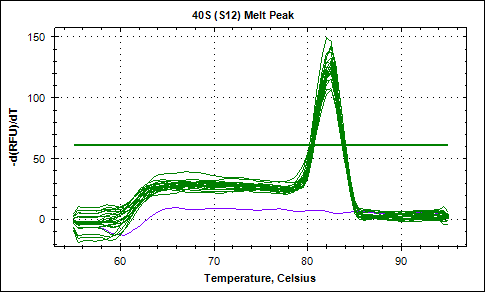


**RSP12 Melt pick**
