# Supplementary material for: Identification and validation of Aeluropus littoralis reference genes for Quantitative Real-Time PCR Normalization
Source: J Biol Res (Thessalon). 2016 Jul 19;23:18. doi: 10.1186/s40709-016-0053-8 (PMC4950632; doi:10.1186/s40709-016-0053-8)
Supplement: Supplementary file 4 — 10.1186/s40709-016-0053-8 The standard curve analysis for calculation PCR efficiency in root (A) and leaf (B) samples. [file 40709_2016_53_MOESM4_ESM.docx]

**Supplementary Figure 4.** The standard curve analysis for calculation PCR efficiency in root (A) and leaf (B) samples.

1. **Root**


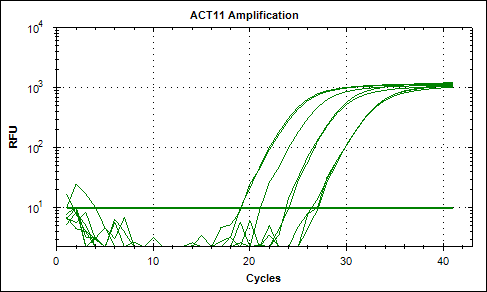

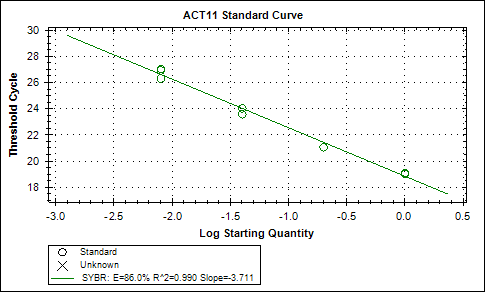


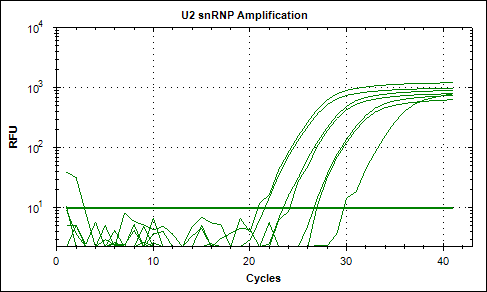

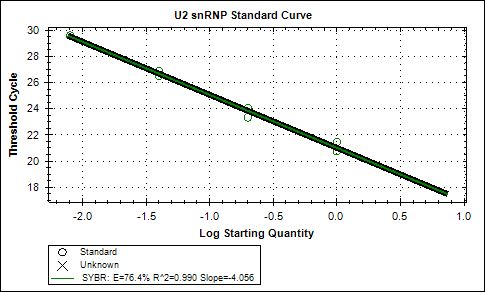


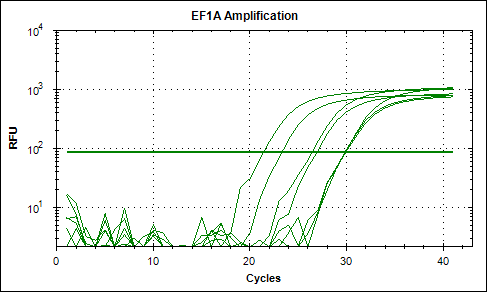

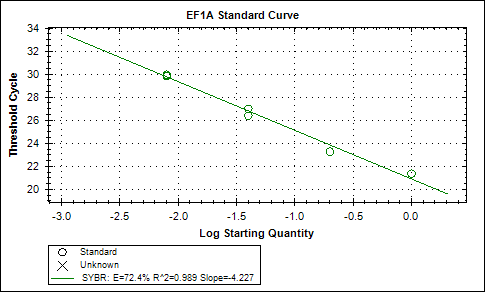


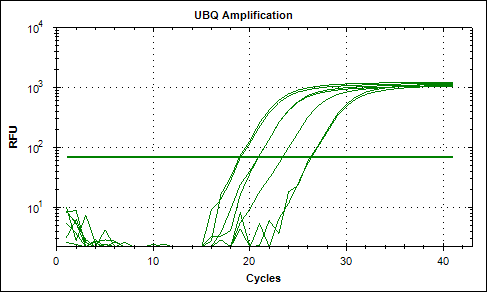

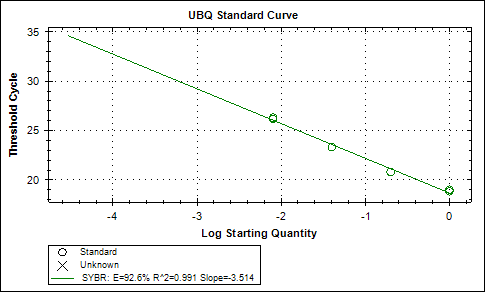


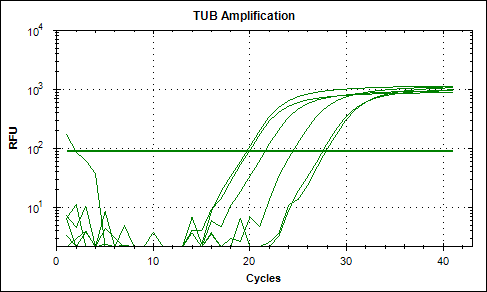

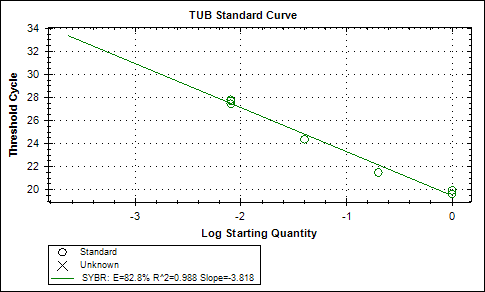


**U2SURP Amplification**

**U2SURP Standard curve**


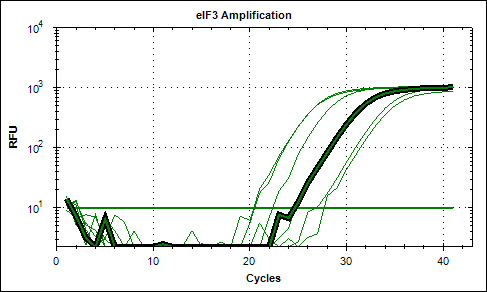

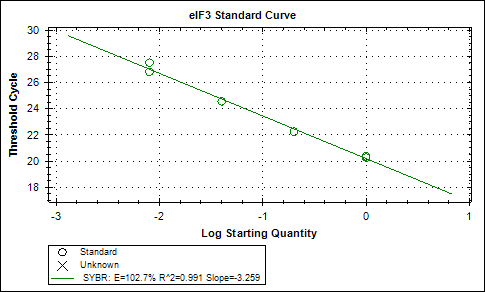


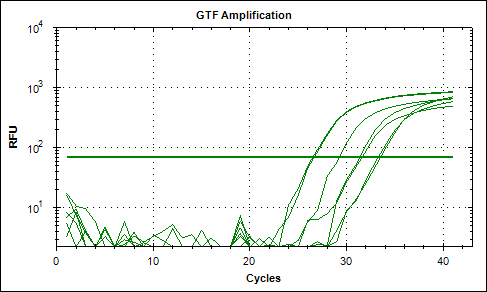

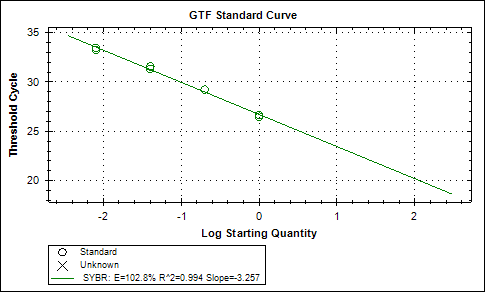


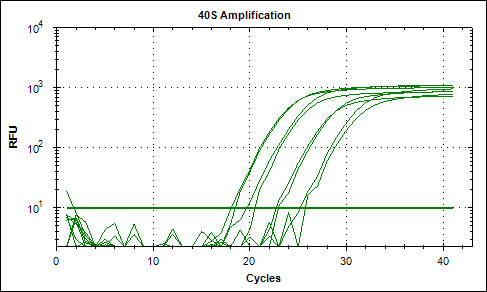

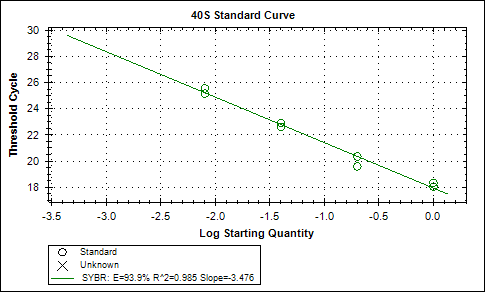


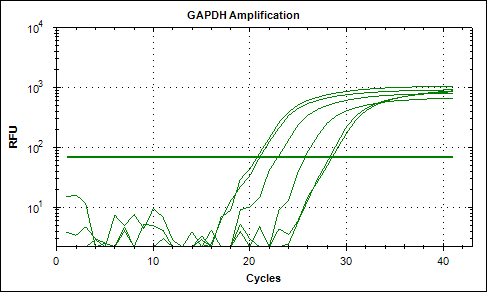

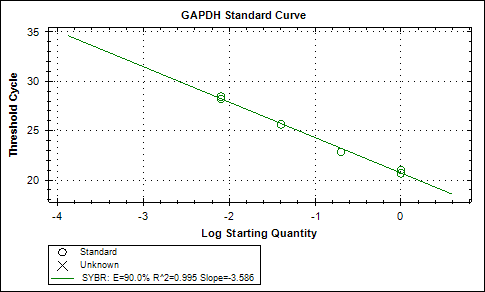


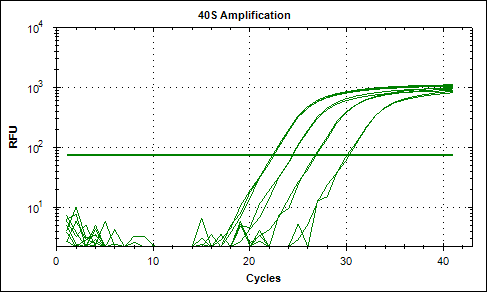

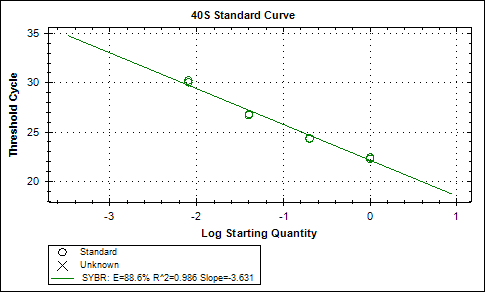


**RPS3 Standard curve**

**RPS3 Amplification**

**RPS12 Standard curve**

**RPS12 Amplification**

1. **Leaf**


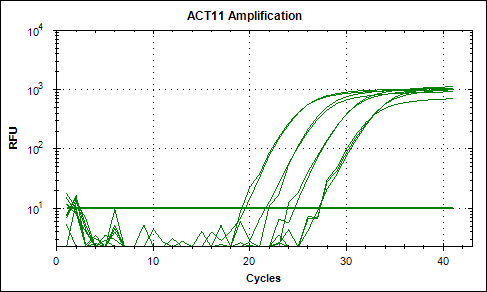

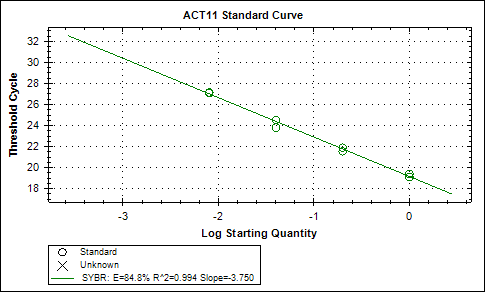


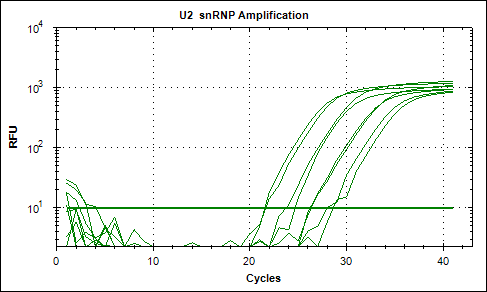

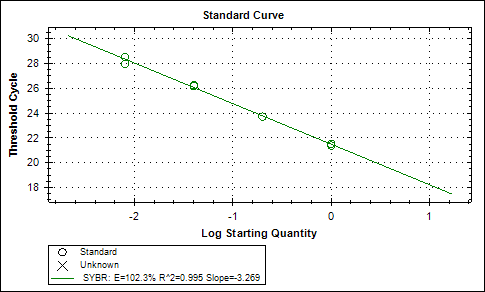


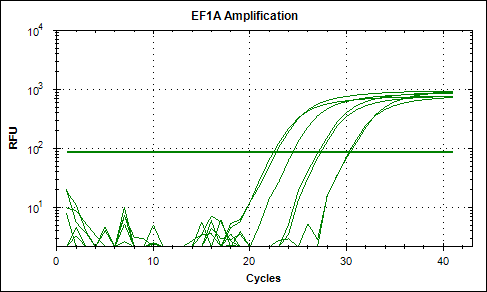

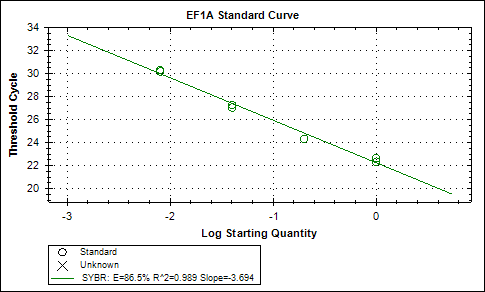


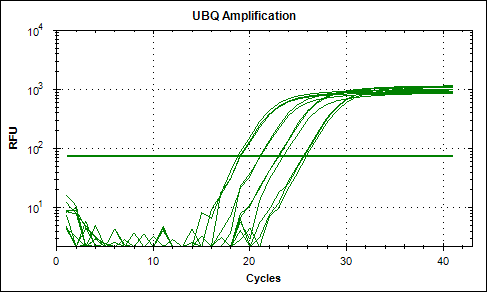

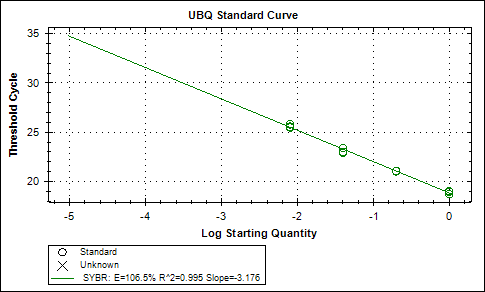


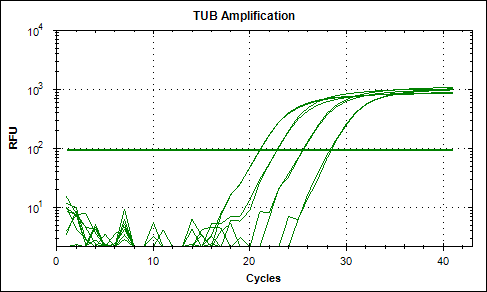

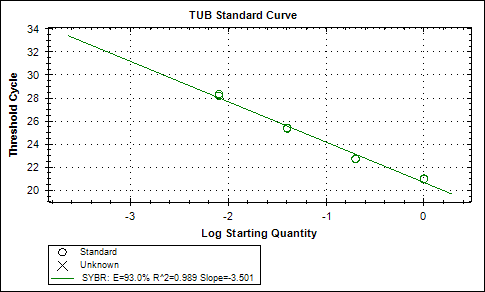


**U2SURP Standard curve**

**U2SURP Amplification**


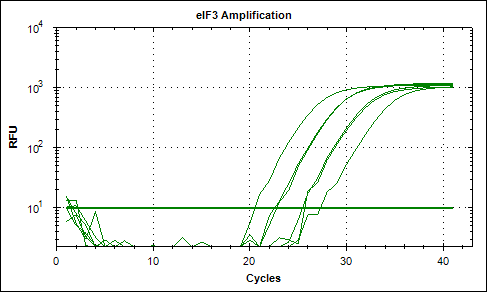

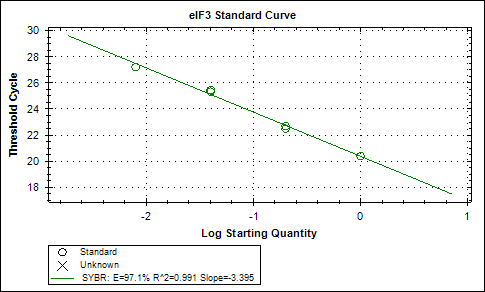


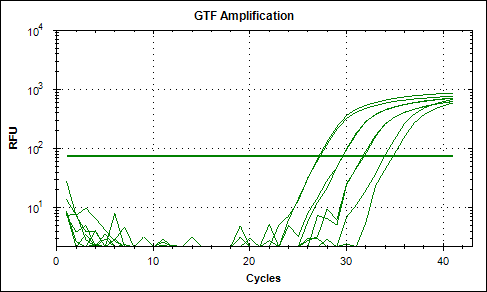

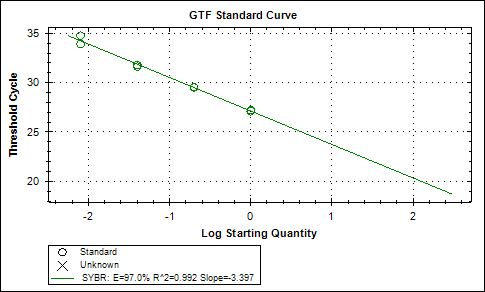


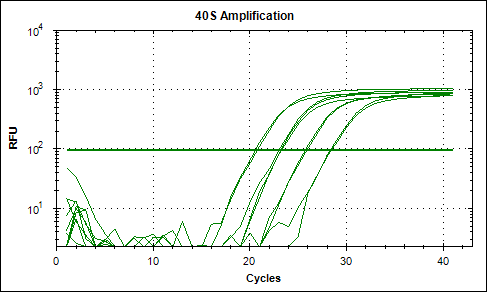

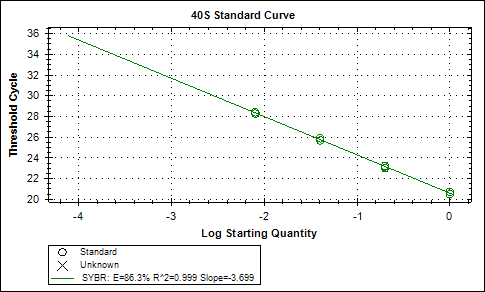


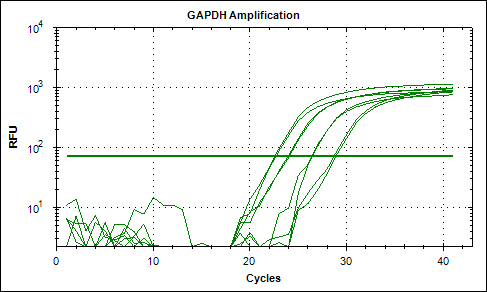

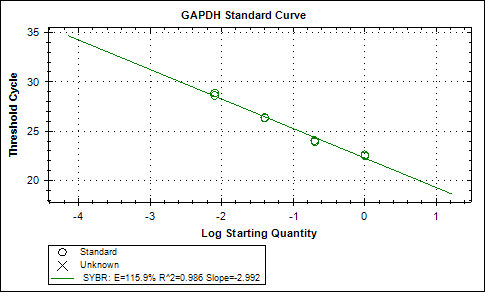


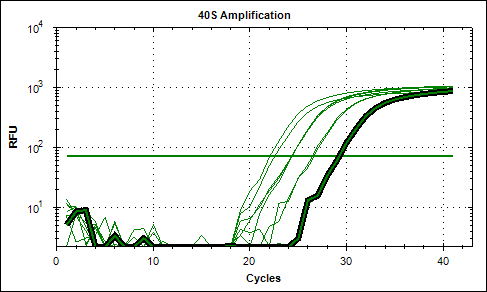

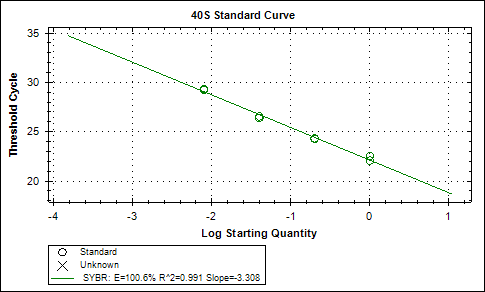


**RPS3 Standard curve**

**RPS3 Amplification**

**RPS12 Standard curve**

**RPS12 Amplification**
