# Supplementary material for: Identification and validation of Aeluropus littoralis reference genes for Quantitative Real-Time PCR Normalization
Source: J Biol Res (Thessalon). 2016 Jul 19;23:18. doi: 10.1186/s40709-016-0053-8 (PMC4950632; doi:10.1186/s40709-016-0053-8)
Supplement: Supplementary file 5 — 10.1186/s40709-016-0053-8 The primer features of candidate reference genes and their PCR efficiencies. [file 40709_2016_53_MOESM5_ESM.docx]

**Supplementary Table S1.** The primer features of candidate reference genes and their PCR efficiencies. Abbreviations: E: Mean PCR efficiency; R^2^: Correlation coefficient; Amp Tm: Amplicon melting temperature; Basel V: Baseline threshold value.

|  | Leaf | | | | | |  | Root | | | | | | Primer length | Melting temperature | GC content | Amplicon size |
| --- | --- | --- | --- | --- | --- | --- | --- | --- | --- | --- | --- | --- | --- | --- | --- | --- | --- |
| Candidate reference genes | E. calculated from calibration curves | R^2^ | E calculated from LinRegPCR | R^2^ | Amplicon Tm | Basel. V |  | E. calculated from calibration curves | R^2^ | E calculated from LinRegPCR | R^2^ | Amplicon Tm | Basel. V |  |  |  |  |
| *ACT11* | 84.4 | 0.994 | 84 | 0.999 | 80 | 460 |  | 86.6 | 0.990 | 89 | 0.999 | 80 | 515 | 21  18 | 63  63 | 47  50 | 118 |
| *U2SURP* | 102.3 | 0995 | 82 | 0.999 | 79 | 498 |  | 76.4 | 0.990 | 88 | 0.998 | 79.5 | 57 | 21  19 | 63  63 | 47  52 | 199 |
| *EF1A* | 86.5 | 0.989 | 9 | 0.998 | 80 | 374 |  | 72.4 | 0.989 | 86 | 0.999 | 80 | 486 | 18  20 | 63  62.5 | 50  45 | 97 |
| *UBQ* | 106.5 | 0.995 | 88 | 0.999 | 80.5 | 397 |  | 92.6 | 0.991 | 87 | 0.999 | 80.5 | 562 | 20  21 | 63  63 | 50  48 | 200 |
| *TUB* | 93 | 0.989 | 97 | 0.999 | 76.5 | 321 |  | 82.8 | 0.988 | 90 | 0.999 | 76.5 | 439 | 18  24 | 63  63 | 50  42 | 109 |
| *eIF3* | 97.1 | 0.991 | 9 | 0.999 | 78.5 | 401 |  | 102.7 | 0.991 | 84 | 0.999 | 78.5 | 370 | 20  21 | 58  56 | 65  57 | 126 |
| *GTF* | 97 | 0.992 | 88 | 0.999 | 77 | 374 |  | 102.8 | 0.994 | 92 | 0.998 | 77 | 353 | 20  20 | 63  63 | 50  55 | 108 |
| *RPS12* | 100.6 | 0.991 | 97 | 0.999 | 82.5 | 365 |  | 88.6 | 0.986 | 88 | 0.999 | 82.5 | 332 | 18  18 | 63.5  63.5 | 55  61 | 147 |
| *GAPDH* | 115.9 | 0.986 | 95 | 0.999 | 85 | 341 |  | 90 | 0.995 | 85 | 0.999 | 83 | 472 | 22  20 | 64  64 | 41  45 | 184 |
| *RPS3* | 86.3 | 0.999 | 106 | 0.999 | 78.5 | 71.4 |  | 93.9 | 0.985 | 92 | 0.998 | 78.5 | 508 | 20  20 | 63  63 | 55  60 | 107 |
