# Supplementary material for: Identification and validation of Aeluropus littoralis reference genes for Quantitative Real-Time PCR Normalization
Source: J Biol Res (Thessalon). 2016 Jul 19;23:18. doi: 10.1186/s40709-016-0053-8 (PMC4950632; doi:10.1186/s40709-016-0053-8)
Supplement: Supplementary file 6 — 10.1186/s40709-016-0053-8 Expression stability analysis by BestKeeper. [file 40709_2016_53_MOESM6_ESM.docx]

**Supplementary Table S2. Expression stability analysis by BestKeeper in A) salt stress, B) recovery condition, (C) root, (D) leaf and (E) all samples (N=16).** Abbreviations: N: number of samples; GM [Cq]: the geometric mean of Cq; AM [Cq]: the arithmetic mean of Cq; Min [Cq] and Max [Cq]: the extreme values of Cq; SD [±Cq]: the standard deviation of the Cq; CV [%Cq]: the coefficient of variance expressed as a percentage on the Cq level; Min [x-fold] and Max [x-fold]: the extreme values of expression levels expressed as an absolute x-fold over- or under-regulation coefficient; SD [± x-fold]: standard deviation of the absolute regulation coefficients

**A)** Salt stress

| n | *RPS3* | *GTF* | *ACT11* | *U2SURP* | *EF1A* | *TUB* | *UBQ* | *GAPDH* | *eIF3* | *RPS12* | BestKeeper |
| --- | --- | --- | --- | --- | --- | --- | --- | --- | --- | --- | --- |
|  | 8 | 8 | 8 | 8 | 8 | 8 | 8 | 8 | 8 | 8 | 8 |
| geo Mean [Cq] | 21.89 | 28.61 | 23.82 | 27.34 | 23.08 | 21.15 | 20.39 | 22.45 | 24.41 | 22.22 | 23.41 |
| ar Mean [Cq] | 21.92 | 28.68 | 23.85 | 27.49 | 23.11 | 21.19 | 20.42 | 22.52 | 24.55 | 22.27 | 23.46 |
| min [Cq] | 20.55 | 27.12 | 22.42 | 25.28 | 22.02 | 19.60 | 19.22 | 20.85 | 22.49 | 21.00 | 22.23 |
| max [Cq] | 24.45 | 32.13 | 25.57 | 32.61 | 25.38 | 23.50 | 22.42 | 25.95 | 29.51 | 25.77 | 26.24 |
| std dev [±Cq] | 0.98 | 1.73 | 0.84 | 2.51 | 1.04 | 0.90 | 0.89 | 1.64 | 2.34 | 1.10 | 1.33 |
| CV [%Cq] | 4.47 | 6.01 | 3.52 | 9.12 | 4.50 | 4.26 | 4.35 | 7.28 | 9.52 | 4.92 | 5.68 |
| min [x-fold] | -2.46 | -2.60 | -2.40 | -3.58 | -1.94 | -2.79 | -2.08 | -2.78 | -3.32 | -2.22 | 2.13 |
| max [x-fold] | 5.58 | 9.51 | 2.98 | 26.08 | 4.26 | 4.70 | 3.59 | 9.41 | 24.04 | 10.15 | 6.09 |
| std dev [± x-fold] | 1.93 | 3.19 | 1.76 | 5.39 | 2.01 | 1.83 | 1.82 | 3.01 | 4.81 | 2.09 | 2.34 |
| coeff. of corr. [r] | 0.917 | 0.982 | 0.905 | 0.988 | 0.993 | 0.898 | 0.930 | 0.985 | 0.987 | 0.875 |  |
| p-value | 0.001 | 0.001 | 0.002 | 0.001 | 0.001 | 0.002 | 0.001 | 0.001 | 0.001 | 0.004 |  |
| Power of HKG [x-fold] | 1.60 | 2.24 | 1.46 | 3.14 | 1.65 | 1.58 | 1.50 | 2.17 | 2.93 | 1.72 |  |

**B) Recovery condition**

| n | *RPS3* | *GTF* | *ACT11* | *U2SURP* | *EF1A* | *TUB* | *UBQ* | *GAPDH* | *eIF3* | *RPS12* | BestKeeper |
| --- | --- | --- | --- | --- | --- | --- | --- | --- | --- | --- | --- |
|  | 6 | 6 | 6 | 6 | 6 | 6 | 6 | 6 | 6 | 6 | 6 |
| geo Mean [Cq] | 21.90 | 28.12 | 24.02 | 26.53 | 23.39 | 20.78 | 19.41 | 22.64 | 23.70 | 22.10 | 23.14 |
| ar Mean [Cq] | 21.92 | 28.14 | 24.06 | 26.55 | 23.40 | 20.82 | 19.42 | 22.66 | 23.71 | 22.12 | 23.15 |
| min [Cq] | 20.85 | 27.11 | 21.86 | 25.04 | 22.49 | 19.10 | 18.59 | 21.42 | 22.49 | 21.14 | 22.50 |
| max [Cq] | 23.41 | 29.03 | 25.88 | 27.59 | 24.31 | 22.66 | 20.32 | 23.87 | 24.57 | 24.06 | 24.34 |
| std dev [±Cq] | 0.76 | 0.71 | 1.27 | 0.89 | 0.54 | 0.98 | 0.51 | 0.65 | 0.53 | 0.82 | 0.62 |
| CV [%Cq] | 3.46 | 2.53 | 5.29 | 3.34 | 2.33 | 4.68 | 2.63 | 2.88 | 2.23 | 3.70 | 2.68 |
| min [x-fold] | -2.02 | -1.92 | -3.86 | -2.51 | -1.77 | -3.03 | -1.67 | -2.18 | -2.13 | -1.87 | 1.50 |
| max [x-fold] | 2.76 | 1.79 | 3.19 | 1.93 | 1.78 | 3.45 | 1.77 | 2.20 | 1.72 | 3.59 | 2.15 |
| std dev [± x-fold] | 1.67 | 1.61 | 2.35 | 1.81 | 1.44 | 1.93 | 1.41 | 1.55 | 1.43 | 1.73 | 1.49 |
| coeff. of corr. [r] | 0.933 | 0.682 | 0.837 | 0.547 | 0.851 | 0.675 | 0.698 | 0.794 | 0.779 | 0.883 |  |
| p-value | 0.007 | 0.136 | 0.038 | 0.260 | 0.032 | 0.141 | 0.123 | 0.059 | 0.067 | 0.020 |  |
| Power of HKG [x-fold] | 2.25 | 1.63 | 2.92 | 1.60 | 1.64 | 2.12 | 1.45 | 1.81 | 1.61 | 2.28 |  |

**C) Root**

| n | *RPS3* | *GTF* | *ACT11* | *U2SURP* | *EF1A* | *TUB* | *UBQ* | *GAPDH* | *eIF3* | *RPS12* | BestKeeper |
| --- | --- | --- | --- | --- | --- | --- | --- | --- | --- | --- | --- |
|  | 8 | 8 | 8 | 8 | 8 | 8 | 8 | 8 | 8 | 8 | 8 |
| geo Mean [Cq] | 21.76 | 28.94 | 23.55 | 27.59 | 23.33 | 20.85 | 20.23 | 23.11 | 24.80 | 22.20 | 23.49 |
| ar Mean [Cq] | 21.79 | 29.00 | 23.59 | 27.73 | 23.36 | 20.89 | 20.27 | 23.17 | 24.92 | 22.25 | 23.54 |
| min [Cq] | 20.55 | 27.11 | 21.86 | 25.04 | 22.14 | 19.10 | 18.86 | 20.85 | 22.49 | 21.00 | 22.23 |
| max [Cq] | 24.45 | 32.13 | 25.57 | 32.61 | 25.38 | 23.50 | 22.42 | 25.95 | 29.51 | 25.77 | 26.24 |
| std dev [±Cq] | 0.96 | 1.57 | 1.18 | 2.38 | 0.99 | 1.10 | 0.96 | 1.35 | 2.15 | 1.13 | 1.29 |
| CV [%Cq] | 4.40 | 5.40 | 5.02 | 8.59 | 4.25 | 5.27 | 4.73 | 5.81 | 8.63 | 5.09 | 5.49 |
| min [x-fold] | -2.21 | -3.28 | -2.94 | -5.05 | -2.09 | -3.07 | -2.36 | -4.01 | -4.09 | -2.12 | 2.22 |
| max [x-fold] | 5.81 | 7.97 | 3.62 | 24.07 | 3.57 | 5.48 | 3.92 | 5.71 | 17.56 | 9.38 | 5.65 |
| std dev [± x-fold] | 1.87 | 2.79 | 2.17 | 4.75 | 1.91 | 2.05 | 1.87 | 2.41 | 4.08 | 2.10 | 2.26 |
| coeff. of corr. [r] | 0.954 | 0.980 | 0.886 | 0.966 | 0.977 | 0.845 | 0.929 | 0.954 | 0.986 | 0.886 |  |
| p-value | 0.001 | 0.001 | 0.003 | 0.001 | 0.001 | 0.008 | 0.001 | 0.001 | 0.001 | 0.003 |  |
| Power of HKG [x-fold] | 1.65 | 2.20 | 1.61 | 3.13 | 1.59 | 1.61 | 1.56 | 1.91 | 2.69 | 1.71 |  |

**D) Leaf**

| n | *RPS3* | *GTF* | *ACT11* | *U2SURP* | *EF1A* | *TUB* | *UBQ* | *GAPDH* | *eIF3* | *RPS12* | BestKeeper |
| --- | --- | --- | --- | --- | --- | --- | --- | --- | --- | --- | --- |
|  | 8 | 8 | 8 | 8 | 8 | 8 | 8 | 8 | 8 | 8 | 8 |
| geo Mean [Cq] | 21.95 | 27.79 | 24.16 | 26.13 | 23.05 | 21.04 | 19.50 | 22.01 | 23.24 | 22.04 | 22.98 |
| ar Mean [Cq] | 21.97 | 27.79 | 24.18 | 26.13 | 23.06 | 21.05 | 19.51 | 22.02 | 23.25 | 22.05 | 22.99 |
| min [Cq] | 21.29 | 27.12 | 23.31 | 25.60 | 22.02 | 19.60 | 18.59 | 21.23 | 22.49 | 21.11 | 22.39 |
| max [Cq] | 23.41 | 29.03 | 25.88 | 27.59 | 24.31 | 22.66 | 20.49 | 23.87 | 24.57 | 24.06 | 24.34 |
| std dev [±Cq] | 0.62 | 0.39 | 0.73 | 0.48 | 0.62 | 0.62 | 0.52 | 0.65 | 0.54 | 0.67 | 0.48 |
| CV [%Cq] | 2.83 | 1.42 | 3.00 | 1.84 | 2.68 | 2.95 | 2.67 | 2.97 | 2.34 | 3.06 | 2.11 |
| min [x-fold] | -1.58 | -1.52 | -1.68 | -1.37 | -1.93 | -2.64 | -1.78 | -1.68 | -1.61 | -1.87 | 1.46 |
| max [x-fold] | 2.73 | 2.19 | 2.86 | 2.42 | 2.24 | 3.00 | 1.87 | 3.45 | 2.34 | 3.94 | 2.41 |
| std dev [± x-fold] | 1.53 | 1.31 | 1.65 | 1.39 | 1.53 | 1.54 | 1.43 | 1.57 | 1.45 | 1.59 | 1.37 |
| coeff. of corr. [r] | 0.952 | 0.785 | 0.889 | 0.823 | 0.829 | 0.915 | 0.370 | 0.891 | 0.956 | 0.880 |  |
| p-value | 0.001 | 0.021 | 0.003 | 0.012 | 0.011 | 0.001 | 0.365 | 0.003 | 0.001 | 0.004 |  |
| Power of HKG [x-fold] | 2.15 | 1.56 | 2.17 | 1.66 | 1.85 | 2.32 | 1.26 | 2.22 | 1.89 | 2.39 |  |

**F) All samples (N=16)**

|  | *RPS3* | *GTF* | *ACT11* | *U2SURP* | *EF1A* | *TUB* | *UBQ* | *GAPDH* | *eIF3* | *RPS12* | BestKeeper |
| --- | --- | --- | --- | --- | --- | --- | --- | --- | --- | --- | --- |
| n | 16 | 16 | 16 | 16 | 16 | 16 | 16 | 16 | 16 | 16 | 16 |
| geo Mean [CP] | 21.86 | 28.36 | 23.85 | 26.85 | 23.19 | 20.94 | 19.86 | 22.55 | 24.01 | 22.12 | 23.23 |
| ar Mean [CP] | 21.88 | 28.40 | 23.88 | 26.93 | 23.21 | 20.97 | 19.89 | 22.60 | 24.09 | 22.15 | 23.26 |
| min [CP] | 20.55 | 27.11 | 21.86 | 25.04 | 22.02 | 19.10 | 18.59 | 20.85 | 22.49 | 21.00 | 22.23 |
| max [CP] | 24.45 | 32.13 | 25.88 | 32.61 | 25.38 | 23.50 | 22.42 | 25.95 | 29.51 | 25.77 | 26.24 |
| std dev [± CP] | 0.80 | 1.10 | 0.95 | 1.58 | 0.82 | 0.88 | 0.75 | 1.16 | 1.37 | 0.90 | 0.90 |
| CV [% CP] | 3.66 | 3.88 | 3.96 | 5.86 | 3.55 | 4.20 | 3.76 | 5.14 | 5.69 | 4.08 | 3.88 |
| min [x-fold] | -2.41 | -2.22 | -3.48 | -3.07 | -2.08 | -3.37 | -2.22 | -2.97 | -2.58 | -2.07 | 1.90 |
| max [x-fold] | 5.71 | 11.21 | 3.54 | 35.34 | 3.98 | 5.40 | 4.98 | 8.78 | 30.92 | 10.85 | 6.81 |
| std dev [± x-fold] | 1.71 | 2.10 | 1.89 | 2.89 | 1.74 | 1.81 | 1.65 | 2.18 | 2.51 | 1.84 | 1.78 |
| coeff. of corr. [r] | 0.89 | 0.95 | 0.76 | 0.94 | 0.94 | 0.80 | 0.83 | 0.93 | 0.96 | 0.87 |  |
| p-value | 0.001 | 0.001 | 0.001 | 0.001 | 0.001 | 0.001 | 0.001 | 0.001 | 0.001 | 0.001 |  |
| Power of HKG [x-fold] | 1.65 | 2.15 | 1.57 | 2.92 | 1.62 | 1.64 | 1.55 | 2.06 | 2.74 | 1.79 |  |
