# Supplementary material for: Identification and validation of Aeluropus littoralis reference genes for Quantitative Real-Time PCR Normalization
Source: J Biol Res (Thessalon). 2016 Jul 19;23:18. doi: 10.1186/s40709-016-0053-8 (PMC4950632; doi:10.1186/s40709-016-0053-8)
Supplement: Supplementary file 7 — 10.1186/s40709-016-0053-8 Analysis of sample integrity by BestKeeper. [file 40709_2016_53_MOESM7_ESM.docx]

**Supplementary Table S3. Analysis of sample integrity and expression stability by BestKeeper.** The removed samples from BestKeeper analysis were highlighted in red color (A). Expression stability analysis after removing two samples with high expression variation: root (B) and all samples (C). Abbreviations: N: number of HKG genes was studied, InVar: intrinsic variance of expression, InVar. [± Cq]: intrinsic variance based on Cq units [± Cq], InVar. [ ± % Cq]: intrinsic variance based on percentage of the mean [± %CP], InVar. [± x-fold]: efficiency corrected intrinsic variation of x-fold.

| Samples | N | BestKeeper | InVar. [± CP] | InVar. [ ± % CP] | InVar. [± x-fold] |
| --- | --- | --- | --- | --- | --- |
| Root-control | 10 | 23.10 | 0.46 | 1.90 | 0.30 |
| Salt-stressed root (6h) | 10 | 22.31 | 0.37 | 1.67 | 0.15 |
| Salt-stressed root (24h | 10 | 26.24 | 1.42 | 5.20 | 9.33 |
| Salt-stressed root (48h | 10 | 26.01 | 1.68 | 6.29 | 12.28 |
| Salt-stressed root (1w) | 10 | 22.23 | 0.56 | 2.33 | 0.22 |
| recovered root (6h) | 10 | 22.50 | 0.54 | 2.11 | 0.22 |
| recovered root (24h) | 10 | 22.56 | 0.89 | 3.89 | 0.41 |
| recovered root (1w) | 10 | 23.38 | 0.61 | 2.72 | 0.38 |
| Leaf-control | 10 | 22.58 | 0.32 | 1.49 | 0.13 |
| Salt-stressed leaf (6h) | 10 | 22.77 | 0.51 | 2.08 | 0.25 |
| Salt-stressed leaf (24h | 10 | 22.39 | 0.42 | 1.84 | 0.17 |
| Salt-stressed leaf (48h | 10 | 22.94 | 0.70 | 2.93 | 0.37 |
| Salt-stressed leaf (1w) | 10 | 22.78 | 0.50 | 2.26 | 0.26 |
| recovered leaf (6h) | 10 | 24.34 | 0.64 | 3.06 | 0.84 |
| recovered leaf (24h) | 10 | 22.51 | 0.59 | 2.53 | 0.26 |
| recovered leaf (1w) | 10 | 23.57 | 0.79 | 3.27 | 0.84 |

**B) Root (N=6)**

| n | *RPS3* | *GTF* | *ACT11* | *U2SURP* | *EF1A* | *TUB* | *UBQ* | *GAPDH* | *eIF3* | *RPS12* | BestKeeper |
| --- | --- | --- | --- | --- | --- | --- | --- | --- | --- | --- | --- |
|  | 6 | 6 | 6 | 6 | 6 | 6 | 6 | 6 | 6 | 6 | 6 |
| geo Mean [Cq] | 21.15 | 27.94 | 22.97 | 26.13 | 22.74 | 20.19 | 19.63 | 22.28 | 23.48 | 21.50 | 22.68 |
| ar Mean [Cq] | 21.15 | 27.95 | 22.99 | 26.15 | 22.75 | 20.20 | 19.64 | 22.30 | 23.49 | 21.51 | 22.68 |
| min [Cq] | 20.55 | 27.11 | 21.86 | 25.04 | 22.14 | 19.10 | 18.86 | 20.85 | 22.49 | 21.00 | 22.23 |
| max [Cq] | 21.78 | 28.99 | 24.75 | 27.47 | 23.60 | 21.14 | 20.32 | 23.31 | 24.29 | 22.36 | 23.38 |
| std dev [±Cq] | 0.38 | 0.58 | 0.72 | 0.81 | 0.51 | 0.57 | 0.39 | 0.82 | 0.42 | 0.41 | 0.37 |
| CV [%Cq] | 1.80 | 2.08 | 3.11 | 3.09 | 2.24 | 2.82 | 1.98 | 3.68 | 1.79 | 1.91 | 1.65 |
| min [x-fold] | -1.50 | -1.71 | -2.01 | -1.96 | -1.46 | -2.05 | -1.62 | -2.50 | -1.86 | -1.39 | 1.33 |
| max [x-fold] | 1.53 | 1.95 | 3.03 | 2.29 | 1.72 | 1.87 | 1.54 | 1.93 | 1.65 | 1.75 | 1.57 |
| std dev [± x-fold] | 1.29 | 1.48 | 1.62 | 1.72 | 1.41 | 1.47 | 1.30 | 1.74 | 1.33 | 1.32 | 1.27 |
| coeff. of corr. [r] | 0.917 | 0.759 | 0.840 | 0.523 | 0.982 | -0.080 | 0.370 | 0.821 | 0.812 | 0.612 |  |
| p-value | 0.010 | 0.080 | 0.036 | 0.286 | 0.001 | 0.881 | 0.469 | 0.045 | 0.050 | 0.196 |  |
| Power of HKG [x-fold] | 1.91 | 2.14 | 3.13 | 2.03 | 2.33 | 0.92 | 1.30 | 3.04 | 1.93 | 1.57 |  |

**C) All samples (N=14)**

|  | *RPS3* | *GTF* | *ACT11* | *U2SURP* | *EF1A* | *TUB* | *UBQ* | *GAPDH* | *eIF3* | *RPS12* | BestKeeper |
| --- | --- | --- | --- | --- | --- | --- | --- | --- | --- | --- | --- |
| n | 14 | 14 | 14 | 14 | 14 | 14 | 14 | 14 | 14 | 14 | 14 |
| geo Mean [Cq] | 21.61 | 27.86 | 23.65 | 26.13 | 22.91 | 20.67 | 19.56 | 22.12 | 23.34 | 21.81 | 22.85 |
| ar Mean [Cq] | 21.62 | 27.86 | 23.67 | 26.14 | 22.92 | 20.69 | 19.56 | 22.14 | 23.35 | 21.82 | 22.86 |
| min [Cq] | 20.55 | 27.11 | 21.86 | 25.04 | 22.02 | 19.10 | 18.59 | 20.85 | 22.49 | 21.00 | 22.23 |
| max [Cq] | 23.41 | 29.03 | 25.88 | 27.59 | 24.31 | 22.66 | 20.49 | 23.87 | 24.57 | 24.06 | 24.34 |
| std dev [±Cq] | 0.54 | 0.48 | 0.81 | 0.62 | 0.59 | 0.68 | 0.47 | 0.74 | 0.51 | 0.59 | 0.44 |
| CV [%Cq] | 2.48 | 1.71 | 3.44 | 2.37 | 2.56 | 3.30 | 2.40 | 3.36 | 2.17 | 2.72 | 1.91 |
| min [x-fold] | -2.03 | -1.61 | -3.05 | -1.96 | -1.76 | -2.81 | -1.83 | -2.26 | -1.70 | -1.69 | 1.49 |
| max [x-fold] | 3.36 | 2.12 | 4.04 | 2.47 | 2.41 | 3.72 | 1.80 | 3.05 | 2.15 | 4.35 | 2.59 |
| std dev [± x-fold] | 1.43 | 1.38 | 1.73 | 1.52 | 1.48 | 1.58 | 1.37 | 1.65 | 1.41 | 1.49 | 1.32 |
| coeff. of corr. [r] | 0.906 | 0.678 | 0.835 | 0.621 | 0.879 | 0.658 | 0.324 | 0.762 | 0.804 | 0.833 |  |
| p-value | 0.001 | 0.008 | 0.001 | 0.018 | 0.001 | 0.010 | 0.257 | 0.002 | 0.001 | 0.001 |  |
| Power of HKG [x-fold] | 2.22 | 1.60 | 2.71 | 1.69 | 1.95 | 1.97 | 1.23 | 2.12 | 1.73 | 2.16 |  |
