# Supplementary material for: Identification and validation of Aeluropus littoralis reference genes for Quantitative Real-Time PCR Normalization
Source: J Biol Res (Thessalon). 2016 Jul 19;23:18. doi: 10.1186/s40709-016-0053-8 (PMC4950632; doi:10.1186/s40709-016-0053-8)
Supplement: Supplementary file 8 — 10.1186/s40709-016-0053-8 Expression stability analysis by geNorm and NormFinder. [file 40709_2016_53_MOESM8_ESM.docx]

**Supplementary Table S4.** Expression stability analysis by geNorm and NormFinder.

1. geNorm

|  | Stability value | | | | |
| --- | --- | --- | --- | --- | --- |
| Gene name | **Salt stress** | **Recovery condition** | **Root** | **Leaf** | **All samples** |
| *RPS3* | 0.411953 | 0.577545 | 0.356548 | 0.455898 | 0.422899 |
| *GTF* | 0.679479 | 0.806418 | 0.837471 | 0.29553 | 0.794763 |
| *ACT11* | 0.733467 | 0.337447 | 0.522158 | 0.508673 | 0.653774 |
| *U2SURP* | 0.975937 | 1.09596 | 1.092982 | 0.29553 | 1.071719 |
| *EF1A* | 0.411953 | 0.337447 | 0.356548 | 0.480667 | 0.422899 |
| *TUB* | 0.63809 | 0.453911 | 0.78389 | 0.494813 | 0.694042 |
| *UBQ* | 0.533643 | 0.642539 | 0.581801 | 0.591624 | 0.736573 |
| *GAPDH* | 0.47198 | 0.745868 | 0.725747 | 0.432599 | 0.601134 |
| *eIF3* | 0.850275 | 1.008766 | 0.951311 | 0.39211 | 0.932769 |
| *RPS12* | 0.593507 | 0.854587 | 0.658695 | 0.530991 | 0.527635 |
| Best genes | *RPS3/EF1A* | *ACT11/EF1A* | *RPS3/EF1A* | *GTF/U2SURP* | *RPS3/EF1A* |

1. NormFinder

|  | Stability value | | | | |
| --- | --- | --- | --- | --- | --- |
| Gene name | **Salt stress** | **Recovery condition** | **Root** | **Leaf** | **All samples** |
| *RPS3* | 0.033 | 0.424 | 0.184 | 0.056 | 0.268 |
| *GTF* | 0.273 | 0.284 | 0.100 | 0.106 | 0.314 |
| *ACT11* | 0.646 | 0.445 | 0.153 | 0.204 | 0.574 |
| *U2SURP* | 0.962 | 0.955 | 0.108 | 0.102 | 1.027 |
| *EF1A* | 0.376 | 0.278 | 0.048 | 0.122 | 0.396 |
| *TUB* | 0.500 | 0.594 | 0.173 | 0.061 | 0.496 |
| *UBQ* | 0.453 | 0.544 | 0.097 | 0.311 | 0.474 |
| *GAPDH* | 0.327 | 0.341 | 0.049 | 0.094 | 0.376 |
| *eIF3* | 0.647 | 0.757 | 0.107 | 0.023 | 0.729 |
| *RPS12* | 0.233 | 0.619 | 0.249 | 0.105 | 0.426 |
| Best gene | *RPS3* | *EF1A* | *EF1A* | *EF1A* | *RPS3* |
