# Supplementary material for: Identification and validation of Aeluropus littoralis reference genes for Quantitative Real-Time PCR Normalization
Source: J Biol Res (Thessalon). 2016 Jul 19;23:18. doi: 10.1186/s40709-016-0053-8 (PMC4950632; doi:10.1186/s40709-016-0053-8)
Supplement: Supplementary file 9 — 10.1186/s40709-016-0053-8 Ten candidate reference genes in order to higher to lower expression stability were ranked by different approaches. [file 40709_2016_53_MOESM9_ESM.docx]

**Supplementary Table S5.** Ten candidate reference genes in order to higher to lower expression stability were ranked by different approaches. The BestKeeper_,_ geNorm and NormFinder are represented by symbol of A, B and C, respectively.

| All samples | | | Leaf samples | | | Root samples | | | Recovery condition | | |  | Salt stress | | |  |
| --- | --- | --- | --- | --- | --- | --- | --- | --- | --- | --- | --- | --- | --- | --- | --- | --- |
| C | **B** | **A** | **C** | **B** | **A** | **C** | **B** | **A** | **C** | **B** | **A** |  | **C** | **B** | **A** | **Ranking** |
| *EF1A* | *RPS3/ EF1A* | *UBQ* | *eIF3* | *GTF/*  *U2SURP* | *GTF* | *EF1A/*  *GAPDH* | *RPS3/ EF1A* | *RPS3* | *EF1A* | *ACT11/*  *EF1A* | *UBQ* |  | *RPS3* | *RPS3/ EF1A* | *ACT11* | 1. |
| *GTF* | *-* | *GTF* | *RPS3* | *-* | *U2SURP* | *-* | *-* | *UBQ* | *GTF* | *-* | *eIF3* |  | *RPS12* | *-* | *UBQ* | 2. |
| *U2SURP* | *RPS12* | *eIF3* | *TUB* | *eIF3* | *UBQ* | *UBQ* | *ACT11* | *RPS12* | *GAPDH* | *TUB* | *EF1A* |  | *GTF* | *GAPDH* | *TUB* | 3. |
| *RPS3* | *GAPDH* | *RPS3* | *GAPDH* | *GAPDH* | *eIF3* | *GTF* | *UBQ* | *eIF3* | *RPS3* | *RPS3* | *GAPDH* |  | *GAPDH* | *UBQ* | *RPS3* | 4. |
| *TUB* | *ACT11* | *RPS12/ EF1A* | *U2SURP* | *RPS3* | *EF1A* | *eIF3/ U2SURP* | *RPS12* | *EF1A* | *ACT11* | *UBQ* | *GTF* |  | *EF1A* | *RPS12* | *EF1A* | 5. |
| *GAPDH* | *TUB* | *-* | *RPS12/*  *GTF* | *EF1A* | *RPS3* | *-* | *GAPDH* | *TUB* | *UBQ* | *GAPDH* | *RPS3* |  | *UBQ* | *TUB* | *RPS12* | 6. |
| *eIF3* | *UBQ* | *U2SURP* | *-* | *TUB* | *TUB* | *ACT11* | *TUB* | *GTF* | *TUB* | *GTF* | *RPS12* |  | *TUB* | *GTF* | *GAPDH* | 7. |
| *RPS12* | *GTF* | *TUB* | *EF1A* | *ACT11* | *GAPDH* | *TUB* | *GTF* | *ACT11* | *RPS12* | *RPS12* | *U2SURP* |  | *ACT11* | *ACT11* | *GTF* | 8. |
| *ACT11* | *eIF3* | *GAPDH* | *ACT11* | *RPS12* | *RPS12* | *RPS3* | *eIF3* | *U2SURP* | *eIF3* | *eIF3* | *TUB* |  | *eIF3* | *eIF3* | *eIF3* | 9. |
| *UBQ* | *U2SURP* | *ACT11* | *UBQ* | *UBQ* | *ACT11* | *RPS12* | *U2SURP* | *GAPDH* | *U2SURP* | *U2SURP* | *ACT11* |  | *U2SURP* | *U2SURP* | *U2SURP* | 10. |
